# The rise of army ants and their relatives: diversification of specialized predatory doryline ants

**DOI:** 10.1186/1471-2148-14-93

**Published:** 2014-05-01

**Authors:** Seán G Brady, Brian L Fisher, Ted R Schultz, Philip S Ward

**Affiliations:** 1 Department of Entomology, National Museum of Natural History, Smithsonian Institution, Washington, D.C., USA; 2 Department of Entomology, California Academy of Sciences, San Francisco, CA, USA; 3 Department of Entomology and Nematology, University of California, Davis, CA, USA

## Abstract

**Background:**

Army ants are dominant invertebrate predators in tropical and subtropical terrestrial ecosystems. Their close relatives within the dorylomorph group of ants are also highly specialized predators, although much less is known about their biology. We analyzed molecular data generated from 11 nuclear genes to infer a phylogeny for the major dorylomorph lineages, and incorporated fossil evidence to infer divergence times under a relaxed molecular clock.

**Results:**

Because our results indicate that one subfamily and several genera of dorylomorphs are non-monophyletic, we propose to subsume the six previous dorylomorph subfamilies into a single subfamily, Dorylinae. We find the monophyly of Dorylinae to be strongly supported and estimate the crown age of the group at 87 (74–101) million years. Our phylogenetic analyses provide only weak support for army ant monophyly and also call into question a previous hypothesis that army ants underwent a fundamental split into New World and Old World lineages. Outside the army ants, our phylogeny reveals for the first time many old, distinct lineages in the Dorylinae. The genus *Cerapachys* is shown to be non-monophyletic and comprised of multiple lineages scattered across the Dorylinae tree. We recover, with strong support, novel relationships among these *Cerapachys*-like clades and other doryline genera, but divergences in the deepest parts of the tree are not well resolved. We find the genus *Sphinctomyrmex*, characterized by distinctive abdominal constrictions, to consist of two separate lineages with convergent morphologies, one inhabiting the Old World and the other the New World tropics.

**Conclusions:**

While we obtain good resolution in many parts of the Dorylinae phylogeny, relationships deep in the tree remain unresolved, with major lineages joining each other in various ways depending upon the analytical method employed, but always with short internodes. This may be indicative of rapid radiation in the early history of the Dorylinae, but additional molecular data and more complete species sampling are needed for confirmation. Our phylogeny now provides a basic framework for comparative biological analyses, but much additional study on the behavior and morphology of doryline species is needed, especially investigations directed at the non-army ant taxa.

## Background

The lack of a robust phylogeny for the specialized predators within the dorylomorph ants has been recognized as a major gap in our current knowledge of higher-level ant diversification [1]. This group comprises six subfamilies and approximately 670 described species, distributed collectively across most major biogeographic realms, with highest diversity in tropical latitudes [2]. Monophyly of the dorylomorphs as a whole was established several decades ago based on a suite of morphological characters [3,4] and has subsequently been verified through phylogenetic analyses of several molecular data sets [5-8]. However, relationships among the major dorylomorph lineages remain almost completely unresolved.

The dorylomorphs include the notorious army ants, well-known for their large colony sizes, nomadic behavior, mass foraging, and strong ecological impacts on communities [9-11]. Army ants do not use individual scouts to locate food, as do most other ant species; rather, they send out swarms of up to several hundred thousand workers *en masse* to kill prey items and transport them back to their colonies. Some army ant species show synchronized reproductive cycles in which all brood in a colony are at the same stage in development. Another feature that separates army ants from many other ant species is the nomadic nesting behavior observed in many aboveground species, in which colonies periodically move their habitation sites (and hence foraging ranges) to new locations. As a result of these foraging characteristics, army ants considerably impact their prey populations both ecologically (by affecting prey abundance) and evolutionarily (by selecting for specialized prey defenses) [10].

In contrast to the relatively well-studied army ants, most other dorylomorph species are less conspicuous ants whose biology remains far less well known. Available information suggests that all dorylomorph ants are specialized predators of social insects, particularly other ant species and termites. However, many epigaeic (hunting aboveground) and some hypogaeic (underground) army ant species have secondarily expanded their diet breadth to include other invertebrates and even small vertebrates [11,12]. Another characteristic of many dorylomorph ants is the presence of permanently wingless queens. Such species, found in many dorylomorph groups including all army ants, do not have winged queens that engage in mating flights as in most other ant species, but rather possess large wingless queens that mate within the nest and then disperse on the ground via colony fission.

The dorylomorphs occupy a key position in the overall phylogeny of ants, forming the sister group to all other formicoids [7,8], a clade first revealed by molecular phylogenetic data, which contains several major lineages (including the Myrmicinae, Formicinae, and Dolichoderinae) and the bulk of ant species diversity (approximately 90% [13]). In contrast to the secure placement of dorylomorphs within the ant phylogeny, the relationships of many taxa within the dorylomorphs have resisted robust phylogenetic resolution. Previous work within the dorylomorphs has focused almost exclusively on the phylogeny of army ants [5,12,14]. These ants – comprising the subfamilies Aenictinae, Dorylinae, and Ecitoninae – are by far the largest and most widespread group to possess the army ant syndrome of traits described above (nomadic colonies, mass group foraging, and highly modified queens) [9,10]. Previous phylogenies of army ants have suggested that the three army ant subfamilies (together with the enigmatic subfamily Aenictogitoninae) form a monophyletic group, with the New World Ecitoninae forming the sister group to the exclusively Old World lineages [5,14]. However, some of these conclusions were not strongly corroborated by subsequent larger-scale ant phylogenies that included fewer dorylomorph taxa but more molecular data [7,8]. The relationships among the non-army ant lineages remain even more obscure, such that the phylogenetic positions of virtually all non-army ant genera and higher-level groups cannot be established from the previous studies. More troubling is the lack of support for the monophyly of several key genera, including the genus *Cerapachys*, which constitutes the majority of species diversity within the non-army ant dorylomorphs.

Here we present the first comprehensive phylogenetic analysis of major dorylomorph lineages. We update hypotheses regarding the evolution of major army ant lineages, and for the first time identify a series of well-supported, distinct lineages among the non-army ant dorylomorphs. Our divergence dating estimates suggest a rapid radiation of major lineages soon after the origin of this group.

## Results and discussion

### Summary of phylogenetic analyses and reclassification of the dorylomorphs

We generated a molecular data set consisting of DNA sequences from 11 genes obtained from 73 dorylomorph taxa and 10 outgroup taxa. We inferred the phylogeny of dorylomorph lineages under several different analytical treatments of the data. Our fundamental analyses included all generated data, analyzed under partitioned Bayesian inference (BI) and maximum likelihood (ML) frameworks. We found evidence of base composition heterogeneity in the third codon positions of all protein-coding genes (Table 1), a condition long known to potentially compromise phylogenetic inference using standard model implementations [15,16]. To address this, we conducted analyses (i) in which all or some third positions were RY-coded, a technique that recodes nucleotides into purines (A or G) or pyrimidines (C or T) to reduce the influence of base compositional bias [17,18]; (ii) in which third positions were excluded; and (iii) in which codon models were applied to the nine protein-coding genes. We also found evidence that four species (*Cerapachys splendens, Sphinctomyrmex stali*, *Tanipone zona, Vicinopone conciliatrix*) acted as “wildcard” taxa, the inferred phylogenetic positions of which varied markedly depending on analytical treatment. Unlike the most commonly encountered wildcard taxa, these four do not contain any missing nucleotide data. Nonetheless, they are able to occupy multiple, often very different positions in the phylogeny with little or no penalty in likelihood or posterior probability, apparently due to high levels of autapomorphic, symplesiomorphic, and/or homoplastic character states and correspondingly low levels of synapomorphies reliably linking them to other, more stable taxa and clades. This property can lead to erosion in support in extended regions of the phylogeny [19,20]. Thus, we also conducted alternative analyses in which these potential wildcard taxa were excluded.

**Table 1:** Sequence characteristics, models of evolution, and base frequency heterogeneity for various character partitions

**Partition**	**Characters**	**Variable nonPI**	**Variable PI**	**Model (AIC) MrModeltest**	**Full Model (AIC) Modeltest**	**BaseFreq Het p value**
abdA	606	29	183	GTR+I+G	K81uf+I+G	0.9999
abdA pos3	202	14	161	GTR+I+G	GTR+I+G	**0.0000**
abdA pos1&2	404	15	22	GTR+I+G	K81uf+I+G	1.0000
EF1aF2	517	5	187	GTR+I+G	TVM+I+G	**0.0132**
EF1aF2 pos3	173	0	165	GTR+I+G	GTR+I+G	**0.0000**
EF1aF2 pos1&2	344	5	22	GTR+I+G	GTR+I+G	1.0000
LWRh	458	28	234	GTR+I+G	TVM+I+G	0.9944
LWRh pos1	153	11	68	GTR+I+G	K81uf+I+G	1.0000
LWRh pos2	152	10	31	GTR+I+G	TVM+I+G	1.0000
LWRh pos3	153	7	135	GTR+I+G	TVM+I+G	**0.0000**
argK	673	25	298	SYM+I+G	TIMef+I+G	0.9511
argK pos1	224	9	55	GTR+I+G	GTR+I+G	1.0000
argK pos2	224	11	28	GTR+I+G	TVM+I+G	1.0000
argK pos3	225	5	215	HKY+G	HKY+G	**0.0000**
Top1	880	48	399	GTR+I+G	GTR+I+G	**0.0000**
Top1 pos1	293	25	68	GTR+I+G	TrN+I+G	1.0000
Top1 pos2	293	21	51	GTR+I+G	GTR+I+G	1.0000
Top1 pos3	294	2	280	GTR+I+G	TVM+I+G	**0.0000**
Ubx	623	42	189	HKY+I+G	K81uf+I+G	0.9999
Ubx pos3	208	25	160	K80+G	K80+I+G	**0.0000**
Ubx pos1&2	415	17	29	GTR+I+G	TIM+I+G	1.0000
EF1aF1	357	14	119	GTR+I+G	TIM+I+G	1.0000
EF1aF1 pos3	119	7	109	HKY+G	K81uf+G	**0.0001**
EF1aF1 pos1&2	238	7	10	GTR+G	TrN+G	1.0000
wg	411	22	223	GTR+I+G	TIM+I+G	1.0000
wg pos1	137	11	53	SYM+I+G	TVMef+I+G	1.0000
wg pos2	137	8	38	GTR+G	GTR+G	1.0000
wg pos3	137	3	132	GTR+G	TIM+G	**0.0000**
CAD	981	62	504		TIM+I+G	**0.0000**
CAD pos1	327	35	114	GTR+I+G	TrN+I+G	1.0000
CAD pos2	327	24	72	GTR+I+G	TIM+I+G	1.0000
CAD pos3	327	3	318	GTR+I+G	TIM+I+G	**0.0000**
28S	1827	176	300		GTR+I+G	1.0000
18S	1870	71	124	GTR+I+G	GTR+I+G	1.0000

“Variable non-PI” indicates the number of non-parsimony-informative (i.e., autapomorphic) and “Variable PI” indicates the number of parsimony-informative variable characters. P-values in bold signify significant levels of base frequency heterogeneity.

**Figure 1:**
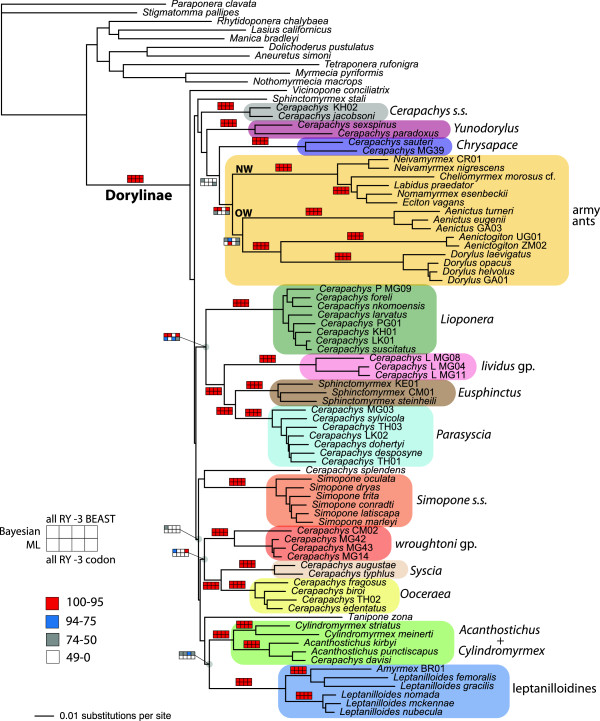
**Summary of phylogenetic results for the dorylines based on analyses of the 83 taxon data set.** The majority rule consensus of all post burnin trees from a Bayesian analysis of the full data set under standard nucleotide coding. The colored boxes above branches denote support values. The top row of each box indicates Bayesian posterior support values, expressed as percentages; the bottom row indicates maximum likelihood (ML) bootstrap values. The columns, from left to right, indicate (i) standard nucleotide coding; (ii) RY-coding of all third codon positions; (iii) exclusion of all third codon positions; (iv) BEAST posterior values under standard coding (top cell), or ML bootstrap values under a codon model (bottom cell). For each cell, white = 0–49, grey = 50–74, blue = 75–94, red = 95–100 (see key).

Under all analytical treatments of the data, the dorylomorphs were inferred to be a robustly supported, monophyletic group (Figure 1 and Additional file 1). This was an expected result in light of the overwhelming support for this clade found in previous morphological and molecular studies [3-8]. The dorylomorphs, as currently defined, encompass six different subfamilies. We find strong support for the monophyly of five of these subfamilies (Aenictinae, Dorylinae, Ecitoninae, Aenictogitoninae, and Leptanilloidinae). However, the sixth subfamily, Cerapachyinae, is heterogeneous and non-monophyletic, comprising all remaining dorylomorphs that cannot be placed in the five more derived groups. The five monophyletic subfamilies are all nested within Cerapachyinae, and no redefinition or subdivision of Cerapachyinae can salvage its monophyly. For this reason, we propose to reclassify all dorylomorph ants as members of a single subfamily to ensure monophyly—a criterion that already applies to all other ant subfamilies except possibly Amblyoponinae [13]. Because Dorylinae represents the oldest available name, it takes priority as the name for all dorylomorphs. Hence, we refer to the dorylomorphs as Dorylinae throughout the remainder of this paper. This establishes the following new synonymy: Dorylinae = Acanthostichini n. syn., = Aenictinae n. syn., = Aenictogitoninae n. syn., = Cerapachyinae (=Eusphinctinae = Lioponerini) n. syn., = Cheliomyrmecini n. syn.,= Cylindromyrmecini n. syn., = Ecitoninae n. syn., = Leptanilloidinae n. syn.

### Army ant evolution

The army ants were reconstructed as monophyletic under most analytical treatments, with support varying considerably depending on treatment conditions. Our BI analysis of the entire data set supports army ant monophyly with a posterior probability (PP) of 0.99 (MrBayes) or 1.0 (BEAST), but ML analyses of this data set only weakly support army ant monophyly under both nucleotide (bootstrap proportion [BP] = 53) and codon (BP = 71) models (Figure 1). The application of RY-coding showed similar trends in support, but with even weaker ML support, while the exclusion of third codon positions yielded essentially no support for this clade. The exclusion of the four wildcard taxa had no appreciable influence on these trends (Additional file 1).

Army ant monophyly would imply a single origin of the army ant syndrome, while non-monophyly would indicate either convergent evolution or an evolutionary reversal of the syndrome. Only the BI analyses yielded strong support for monophyly of this group, and this occurs only when fast evolving third positions are included. Furthermore, the four major army ant clades are all composed of relatively long subtending branches (Figure 1) and it is possible that the strong BI support for army ant monophyly, depending largely on third positions, is an analytical artifact. Thus, evidence for army ant monophyly cannot be considered strong. It should be noted, however, that in the few cases in which army ant monophyly was not recovered, the alternatives were neither well supported nor consistent (Additional file 2).

Our analyses also call into question a fundamental split between Old World and New World army ant lineages, as suggested by previous phylogenies [5,14]. Specifically, support values supporting the monophyly of the Old World army ants (*Aenictus*, *Aenictogiton*, *Dorylus*) are weak in our BI (PP = 0.59 in MrBayes; PP = 0.69 in BEAST) and ML (BP < 50) analyses of the entire data set. A few of our secondary analyses result in stronger support, such as RY-coding BI analyses of the entire data set (PP = 0.84) and with the four wildcard taxa excluded (PP = 0.91); but, all analyses considered, our data provide weak support. It is true that previous studies included more species of army ants, although they also included less molecular data. Future studies including both more taxa and data will be necessary to resolve this issue.

Within the putative Old World army ants, *Aenictogiton* is always reconstructed as the sister to *Dorylus* with overwhelming support. This result accords with previous morphological [14] and molecular [7] phylogenies that also associate these two taxa. Behavioral observations of *Aenictogiton* workers are lacking, and thus we do not know if they possess the same syndrome of behavioral traits shared by all army ants. However, the phylogenetic position of *Aenictogiton* as the sister to *Dorylus*, combined with the fact that no army ant lineage is known to have lost the army ant syndrome, reinforces the prediction that the biology of this group conforms to the army ant syndrome.

Within the New World army ants, the genus *Neivamyrmex* is reconstructed as the sister to all remaining genera including *Cheliomyrmex*. This relationship has been suggested, but without strong support, by previous morphological [14] and molecular [5] data sets that sampled all genera of New World army ants. The rarely encountered genus *Cheliomyrmex* (and not *Neivamyrmex*) has long been considered to retain primitive morphological and perhaps even behavioral characteristics within army ants as a whole [10,21,22]. Its phylogenetic position as reconstructed in our analyses renders problematic any interpretation in which *Cheliomyrmex* represents the ancestral army ant condition [14].

Identifying the sister group to army ants would provide valuable context for understanding the origin and early evolution of the army ant syndrome. Our current data are not able to establish this relationship, as various doryline taxa cluster with the army ants depending on the treatment of the data, though always with poor support (Additional file 1). However, our analyses do indicate that some taxa possessing certain traits in common with army ant species are not in fact closely related to the true army ants (Figure 1). Species within the exclusively Neotropical leptanilloidine clade [23-26], which now includes the genus *Amyrmex* known only from males [27], display some traits similar to army ants including colony emigration and modified queens [23,25,28]. The leptanilloidines, some *Cerapachys* species such as *C. biroi*[29], and some *Sphinctomyrmex* species display highly synchronized brood cycles, also present in some army ant species. All studied non-army ant dorylines appear to use scouts to initiate the raiding of ant nests, unlike army ant mass foraging; but some doryline species do relocate their nests frequently and carry their brood slung underneath their bodies, behaviors reminiscent of army ants (for further discussion and citations, see ref. 11). We caution, however, that essentially nothing is yet known about the biology of many doryline species, preventing detailed analysis of the evolutionary histories of these traits.

### Diversification of non-army ant lineages

The genera *Cylindromymex* and *Acanthostichus* were robustly resolved as sister groups under all analytical treatments. These results are consistent with previous studies [5-8], with one notable caveat. The species *Cerapachys davisi* is clustered within *Acanthostichus* with 100 percent support under all data treatments. *Cylindromymex*, *Acanthostichus*, and *C. davisi* are exclusively New World taxa [30,31]; where their biology is known, they appear to be specialized predators of termites [30,32,33]. *C. davisi* is only known from males, which may explain why this species was not previously recognized as a member of the genus *Acanthostichus*. It is here formally transferred to that genus (*Acanthostichus davisi* n. comb.).

All included *Simopone* species form a monophyletic group. This largely arboreal genus is rarely collected and remains one of the poorest known Dorylinae groups both in terms of taxonomy and basic natural history. Recent revisionary work has begun to remedy the former lacunae, in which a new genus was identified (*Vicinopone*) as distinct from *Simopone* and a second genus newly described (*Tanipone*) from the Old World tropics [34]. Our phylogenetic analyses confirm the distinctness of these two new genera, with neither *Tanipone* nor *Vicinopone* closely related to *Simopone s.s*.

**Figure 2:**
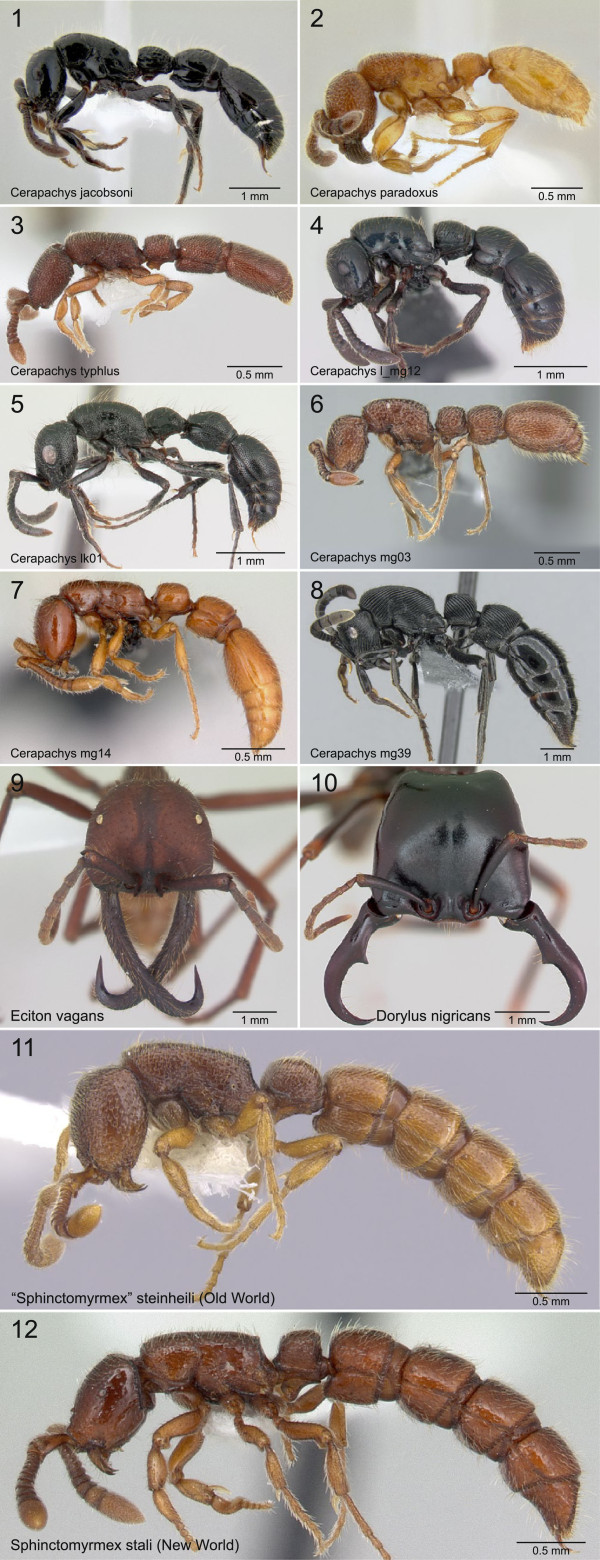
**Morphological diversity within Dorylinae. 1–8**, Lateral views of select *Cerapachys* species representing variation in a generalized morphology that probably reflects the doryline ancestral condition; **9–10**, Dorsal head views of New World (*Eciton*) and Old World (*Dorylus*) army ant species illustrating some morphological characteristics such as falcate mandibles that are linked to their highly specialized foraging behavior; **11–12**, Lateral views of New World and Old World *Sphinctomyrmex* species showing convergent evolution of distinctive abdominal constrictions.

Our analyses strongly indicate the non-monophyly of *Sphinctomyrmex*. This genus is characterized by a diagnostic series of distinct constrictions on the gastral (abdominal) segments (Figure 2). The Old World *Sphinctomyrmex* clade is nested within an Old World group of *Cerapachys* lineages that excludes the New World *S. stali*. Because *S. stali* is the type species of *Sphinctomyrmex*, we informally use the next oldest available name *Eusphinctus* (whose type species is *S. furcatus*, an Asian species) to refer to the Old World clade. Until recently *S. stali* was the only *Sphinctomyrmex* species known from the New World; two more have now been described [35]. The phylogenetic separation of New World and Old World members of the group provides a newly discovered and striking example of morphological convergence. It is possible that these gastral constrictions represent independent adaptations to allow greater dexterity and movement of the stinging apparatus for protection or for stinging prey.

It has long been suspected that the genus *Cerapachys* is not monophyletic. Species within this relatively large genus (147 described species and many more yet to be described; see below) show considerable variation in morphological features, especially with regard to abdominal characters [36], and do not share any reliable morphological characters that might represent putative synapomorphies [4]. Previous molecular phylogenies that included multiple *Cerapachys* species have failed to find support for their monophyly [5,7], although support for non-monophyletic alternative groupings was weak. Our detailed phylogeny now demonstrates without doubt that *Cerapachys* is not monophyletic. More importantly, our results reveal for the first time phylogenetic structure for *Cerapachys* by identifying a series of independent lineages formerly assigned to the genus (Figure 1) as well as establishing some relationships among these lineages. Some of these reconstructed lineages correspond approximately to former genera that were synonymised under *Cerapachys* in the past [32]. For example, we recover with strong support a clade of “*Cerapachys*” species with marked lateral margination on the petiole (*Cerapachys foreli*, *C. larvatus*, *C. suscitatus* and others), for which the genus name *Lioponera* is available. *Lioponera* is sister to a well supported clade that comprises three robust lineages: the “*Cerapachys*” *lividus* group, endemic to Madagascar; the Old World “*Sphinctomyrmex*”, i.e., *Eusphinctus*; and a clade of “*Cerapachys*” species corresponding to the old genus *Parasycia*. Elsewhere in the phylogeny, species assigned to *Cerapachys* fall out as follows: (1) *Cerapachys splendens* is an isolated Neotropical species for which no generic name is available; (2) the erstwhile genus *Yunodorylus*, represented in our study by *C. sexspinus* and *C. paradoxus*, is an independent clade; (3) the erstwhile genus *Chrysapace*, represented by *C. sauteri* and an undescribed Malagasy species, is an independent clade; (4) the true *Cerapachys* (represented by *C. jacobsoni* and *C.* KH02) is distinct, taxonomically isolated, and species-poor; (5) there is a clade of Afrotropical “*Cerapachys*” morphologically similar to *C. wroughtoni* (*C.* CM02, *C.* MG42, *C.* MG43, *C.* MG14); and (6) there are two groups of species with small eyes and reduced body size that are one another’s closest relatives and that correspond to the former genera *Syscia* (e.g., *C. augustae*, *C. typhlus*) and *Ooceraea* (*C. fragosus*, *C. biroi*, *C. edentatus* and others). Thus, our molecular phylogenetic results provide strong justification for reclassifying the unwieldy genus *Cerapachys* into a series of coherent monophyletic groups, providing the basis for future taxonomic work on these groups. We use the erstwhile names (*Chrysapace*, *Syscia*, *Ooceraea*, etc.) here for communication purposes, but we are not proposing formal nomenclatural changes.

We refer to the group containing *C. sexspinus* as “*Yunodorylus*” because this species was originally described as the new genus *Yunodorylus* and was considered to fall within the army ant subfamily Dorylinae *sensu stricto*[37]. Later taxonomic work transferred *Yunodorylus* to *Cerapachys*[4] and described additional species [38]. Our analyses confirm earlier phylogenies [7,8] separating *Yunodorylus* from *Dorylus* and other army ants. One of those earlier phylogenies [7] did indicate, with weak support, that *Yunodorylus* still falls within the army ants, but this position for *Yunodorylus* never resulted from any analytical treatments of the current data set.

### Divergence times and trends within Dorylinae

**Figure 3:**
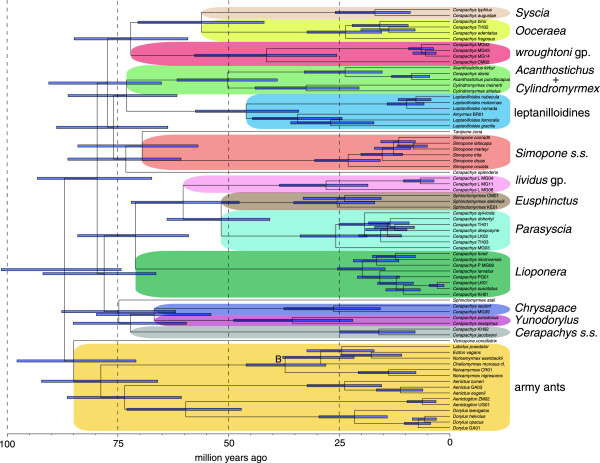
**Chronogram of major lineages within Dorylinae.** Fossil-calibrated chronogram of the Dorylinae inferred under an uncorrelated lognormal relaxed clock model. Branch lengths are proportional to time (in units of millions of years) and horizontal blue bars indicate the 95% highest posterior density of estimated node ages. Support values for this topology are summarized in Figure 1 and Additional file 1.

Our divergence dating analysis indicates that the crown group (i.e., extant) Dorylinae originated approximately 87 (95% CI: 74–101) million years ago (mya) (Figure 3). Initial estimates for the age of this group from previous studies were substantially older, with mean ages of 120 mya [5] and 99–117 mya [8]. Notably, both of these studies contained a large amount of mitochondrial DNA data (cytochrome oxidase I) which can bias divergence dating studies toward older dates [39-41]. Other studies that instead used exclusively nuclear molecular data gave a younger date for Dorylinae, with mean estimates of 77–87 mya [7] using the semiparametric method r8s and 78–82 mya [42] using the same Bayesian method (BEAST) employed in this study. Although these mean ages based on nuclear DNA data do fall within the 95% confidence intervals of our study, some of the discrepancy in mean age estimates between our study and previous work may be due to the relatively poor fossil record for Dorylinae compared to other ant groups. We were able to calibrate only three nodes within the Dorylinae, and the placement of these calibrations may be subject to taxonomic and phylogenetic error. A future goal of divergence dating studies in this group should be to develop comprehensive morphological data sets for both extant and extinct taxa. New methods could then be used that simultaneously infer phylogeny and divergence dates using a combined morphological and molecular character matrix [43-45].

We infer an origin of the crown group army ants at approximately 79 (95% CI: 66–92) mya, about eight million years after the rise of Dorylinae. After this origin, there appears to be a long period without additional speciation (as reflected in extant species) until the crown group origins of each of the four major army ant lineages (*Aenictus*, *Dorylus*, *Aenictogiton*, New World army ants) between 37 and 6 million years ago. It is possible that these long branches are at least partially due to unsampled extant basally diverging lineages in *Aenictus* and *Aenictogiton*, although this explanation remains unlikely for *Dorylus* and New World army ants because they have been the focus of more comprehensive taxonomic and phylogenetic attention [12,46]. The presence of long branches in chronograms of extant taxa is often attributed to one of two factors: a long diversification fuse or massive extinction events. A long diversification fuse would be caused by a long period of very low diversification followed by a rapid increase in diversification rate, while the alternative invokes a period of high levels of extinction rates producing ghost lineages that remain unsampled in a tree of only extant lineages [47,48]. In the absence of a good fossil record, distinguishing between the two scenarios remains difficult even using the most sophisticated diversification methods [49]. Indeed, the present state of Dorylinae systematics precludes the rigorous application of objective diversification rate methods that require estimates of total species diversity within each major lineage. It is true that some smaller taxa within Dorylinae have been the focus of recent taxonomic treatments, including *Acanthostichus*[30], *Cylindromyrmex*[31], *Simopone*[34], and the leptanilloidines [23,25], while the army ants have also been the subjects of taxonomic work by a variety of researchers over the past several decades. But the glaring lacunae in the systematics of the group fall within the large genus *Cerapachys*, which, as our study reveals, embodies most of the taxa involved in the initial diversification of the group. The major lineages in our phylogeny currently assigned to *Cerapachys* contain large numbers of undescribed species (e.g., the taxa in Figure 1 with country codes). Comprehensive systematic treatment of all these lineages currently listed as *Cerapachys* will be necessary to achieve reasonable estimates for lineage species diversity within these groups.

In contrast to the long branches leading to the major crown lineages of Dorylinae, the internodes connecting many of the lineages near the base of the tree are quite short (Figure 1). Our inability to resolve basal diversification within Dorylinae is probably linked to these short branches. Although a lack of data could be responsible for this situation, our matrix includes 3,249 variable sites distributed among 11 nuclear genes, and no taxon in our matrix was missing any sequence data. Rather than an artifact of too little data, these short internodes could instead accurately reflect a rapid radiation of specialized predators. The presence of short basal internodes in our phylogeny also strongly suggests that data of much higher quantity and/or quality, such as those afforded by phylogenomic approaches, will be required to resolve the initial diversification events within Dorylinae.

## Conclusions

Our molecular phylogeny allows us to define major lineages within Dorylinae for the first time. We show that the large genus *Cerapachys* is not monophyletic but instead constitutes about ten distinct lineages, many of which appear to have diversified within a short window of time. The genus *Sphinctomyrmex* resolves as two separate clades, one Old World and the other New World, implying convergent evolution of the abdominal constrictions characteristic of the group.

Our phylogenetic results are valuable not only for encouraging new systematic and taxonomic work on the group, but also for providing a framework for the comparative biological study of these specialized predators. One goal of future work should be to increase our knowledge of predatory behavior, queen mating and dispersal, and other aspects of doryline biology that may have influenced their rates of diversification.

When viewed from the perspective of morphological evolution, it is now evident that taxa presently defined as *Cerapachys* are grouped together not due to unique common descent, but rather because collectively they retain a similar, generalized morphology. In contrast, some other doryline groups, which evolved within the broad assemblage of *Cerapachys*-like forms, acquired distinctively derived morphological features. This includes army ants (e.g., mandible morphology) and the two convergent lineages of *Sphinctomyrmex* (e.g., abdominal constrictions) (Figure 2). Thus, our finding of *Cerapachys* paraphyly is not merely a simple error of taxonomy. We hypothesize that this situation also reflects substantial heterogeneity of evolutionary rates, with taxa that experienced divergent morphological evolution being nested within larger groups that exhibit much greater morphological stasis.

## Methods

### Taxon sampling

We selected for DNA sequencing exemplar taxa that represent the currently known morphological, biogeographical, and taxonomic diversity of the doryline group. Because army ants have already been the focus of several molecular phylogenetic studies [5,12], we focused our sampling on the non-army ant members of Dorylinae (we redefine dorylomorphs as Dorylinae, see Results above). We included a total of 73 Dorylinae species. We used as outgroups members of other formicoid subfamilies and we rooted trees using more distantly related “poneroid” species. Specimens included in this study were collected in accordance with local regulations and all necessary permits were obtained. Ant samples used in this study comply to the regulations for export and exchange of research samples outlined in the Convention of Biology Diversity and the Convention on International Trade in Endangered Species of Wild Fauna and Flora. For field work conducted in Madagascar, permits to research, collect and export ants were obtained from the Ministry of Environment and Forest as part of an ongoing collaboration between the California Academy of Sciences and the Ministry of Environment and Forest, Madagascar National Parks and Parc Botanique et Zoologique de Tsimbazaza. Authorization for export was provided by the Director of Natural Resources. Specimen voucher information is found in Additional file 3.

### DNA sequence generation and alignment

DNA was extracted from single ant specimens using Qiagen kits following the manufacturer’s protocol. In cases in which multiple individuals were available from the same colony, entire or partial specimens were destructively extracted; in cases in which only a single or a few specimens were available, specimens were non-destructively extracted. We sequenced fragments from 11 nuclear genes, including nine protein-coding genes – long wavelength rhodopsin (LWRh), elongation factor 1-alpha F1 copy (EF1aF1), elongation factor 1-alpha F2 copy (EF1aF2), abdominal-A (abdA), wingless (wg), arginine kinase (argK), rudimentary (CAD), ultrabithorax (Ubx), DNA topoisomerase 1 (Top1) – and two ribosomal genes, 18S rDNA and 28S rDNA, for a total of ~9.2 kb of aligned sequence excluding hypervariable regions of 28S (405 sites) and CAD (30 sites) and all introns. Our data matrix (83 taxa by 11 genes) contains no missing fragments. Of the 913 total sequences, 203 were previously published [7,50]; the remaining 710 sequences were generated for the current study. Primer sequences for PCR amplification and sequencing procedures are described in previous publications [7,50-53]. Sequences were collated in Sequencher v4.6 (Gene Codes Corporation), aligned with Clustal X v1.81 [54], and manually edited with MacClade v4.08 [55]. Alignment was straightforward for the exons of protein-coding genes and for 18S. In contrast, the introns of protein-coding genes, hypervariable regions of 28S, and a short region in CAD with many codon triplet indels proved difficult or impossible to align with confidence, and were thus excluded from consideration in all analyses. A list of included taxa with GenBank accession numbers is found in Additional file 3. Sequence characteristics for each gene are summarized in Table 1.

### Data partitions

In most Bayesian and all maximum likelihood (ML) nucleotide-sequence analyses, we partitioned the 11-gene data set into 25 partitions based on the variability of codon-position sites within each gene. Each of four genes (abdA, EF1aF1, EF1aF2, Ubx) were divided into two partitions consisting of (i) codon positions 1 and 2 and (ii) codon position 3, resulting in eight partitions; five genes (LWRh, argK, Top1, wg, CAD) were assigned site-specific models in which each codon position formed a separate partition, resulting in 15 partitions; and two non-protein-coding genes (18S, 28S) were each assigned a single partition, resulting in two partitions. Treatments in which third positions were excluded resulted in 9 fewer partitions and codon-model analyses consisted of 11 partitions, with the nine protein-coding genes analyzed under codon models and 18S and 28S under nucleotide models. The choice of nucleotide substitution model for each partition (Table 1) was determined using the Akaike Information Criterion (AIC) [56] as implemented in ModelTest v3.7 [57] and MrModeltest v3.06 [58].

### Treatments addressing base frequency heterogeneity

For each data partition we evaluated the homogeneity of base frequencies across taxa using PAUP* 4.0b10 [59], which indicated that nine partitions, the third positions of each protein-coding gene, contained significantly heterogeneous base frequencies (Table 1 and Additional file 4). In order to examine the effects of base frequency heterogeneity on phylogenetic results, we employed RY-coding to recode nucleotides as purines (R, i.e., A or G) or pyrimidines (Y, i.e., C or T) to reduce the influence of this compositional bias [17,18]. We subjected each data set to a number of treatments in which the coding of third positions was varied, including: (i) “ACTG,” in which the base identities of codons at third positions were preserved (indicated by “all” in Figure 1); (ii) “partial RY,” in which the subset of taxa (varying according to gene) for which heterogeneous third positions deviated significantly from the observed averages were coded as RY and the remainder of third positions were coded normally (ACTG); (iii) “all RY,” in which all third positions for all taxa and all protein-coding genes were coded as RY; (iv) “excluded,” in which third positions for all taxa and all protein-coding genes were entirely excluded (indicated by “-3” in Figure 1); and (v) codon-model analyses. For treatment ii, “partial RY,” decisions about whether to code a particular third-position site as ACTG or RY were made based on analyses of output from PAUP* as summarized in Additional file 4. Characters were recoded as RY in Mesquite [60].

### Treatments addressing wildcard taxa

In preliminary analyses, a subset of taxa (*Cerapachys splendens, Sphinctomyrmex stali*, *Tanipone zona, Vicinopone conciliatrix*) behaved as “wildcard” taxa whose inferred phylogenetic positions varied substantially depending on the analytical treatment. To explore the impact of including and excluding these four wildcard taxa, we created and analyzed multiple data sets that included: (i) 83 taxa, the full complement; (ii) 79 taxa, from which the four putative “wildcard” taxa were excluded; (iii) 73 taxa, including only the Dorylinae ingroup; (v) 69 taxa, excluding both the non-Dorylinae outgroups and the four wildcard taxa; and (vi) 68 taxa, from which the army ants were excluded.

### Bayesian phylogenetic analyses

We conducted Bayesian analyses using MrBayes v3.1.2 [61] with nucmodel = 4by4, nruns = 2, nchains = 8, and samplefreq = 100, 200, 400, or 1000 depending on the number of generations. For partitioned analyses all parameters, including branch-length rate multipliers, were unlinked across partitions; the only exceptions were branch lengths and topology, which were linked. All analyses were carried out using parallel processing (one chain per CPU) on networked Apple computers with Intel processors. To address known problems with branch-length estimation in MrBayes [62], we set brlenspr = unconstrained: Exp(100); increased the proposal rate from 1.000 to 10.000; and decreased the Dirichlet alpha parameter from 500 to 250 for the rate multipliers (“props” option 26 in MrBayes). Except in the single-gene analyses, we applied moderately informative Dirichlet priors to the rate multipliers to reflect our prior expectation that 28S evolves more rapidly than 18S, and that, for coding regions, third positions evolve faster than first positions, which evolve faster than second positions. Burn-in, convergence, and stationarity were assessed using Tracer v1.5 [63] by examining PSRF values in MrBayes .stat output files, and by using Bayes factor comparisons of harmonic-mean marginal likelihoods of pairs of runs with standard error estimated using 1000 bootstrap pseudoreplicates in Tracer, which employs the weighted likelihood bootstrap estimator of Newton et al. [64] as modified by Suchard et sl. [65]. The results reported here are based on the combined post-burnin data from the two runs that achieved the greatest marginal likelihoods, as summarized in Table 2.

**Table 2:** Summary of Bayesian analyses

**Bayesian analytical treatment**	**Run**	**Taxa**	**Position 3**	**Gens**	**Burn-in**	**PBI gens**	**TracerHM (boots) (best run)**	**Error**	**ln Bayes Factor**
All taxa, Pos3 = ACTG	1	83	ACTG	100M	50M	50M	−101022.705	0.473	1.601
	2	83	ACTG	100M	50M	50M	−101024.305	0.231	−1.601
All taxa, Pos3 = allRY	1	83	allRY	100M	10M	90M	−55074.421	0.192	8.591
	2	83	allRY	100M	10M	90M	−55083.012	0.198	−8.591
All taxa, Pos3 = someRY	1	83	someRY	100M	50M	50M	−83626.678	0.203	−0.646
	2	83	someRY	100M	50M	50M	−83626.032	0.429	0.646
All taxa, Pos3s excluded	1	83	excluded	50M	5M	45M	−40654.165	0.157	0.031
	2	83	excluded	50M	5M	45M	−40654.196	0.148	−0.031
4 wildcards excluded, Pos3 = ACTG	1	79	ACTG	100M	20M	80M	−95642.257	0.871	8.897
	2	79	ACTG	100M	80M	20M	−95651.154	1.21	−8.897
4 wildcards excluded, Pos3 = allRY	1	79	RY	100M	60M	40M	−52539.793	0.294	5.542
	2	79	RY	100M	60M	40M	−52545.335	0.323	−5.542
4 wildcards excluded, Pos3 = someRY	1	79	someRY	100M	50M	50M	−78967.945	0.387	0.748
	2	79	someRY	100M	50M	50M	−78968.693	0.337	−0.748
4 wildcards excluded, Pos3s excluded	1	79	excluded	50M	5M	45M	−38944.878	0.173	−0.214
	2	79	excluded	50M	5M	45M	−38944.664	0.181	0.214
Ingroup only, Pos3 = ACTG	1	73	ACTG	50M	20M	30M	−82585.121	0.32	4.042
	2	73	ACTG	50M	20M	30M	−82589.162	0.41	−4.042
Ingroup only, Pos3 = allRY	1	73	RY	50M	20M	30M	−45906.942	0.289	−7.406
	2	73	RY	50M	20M	30M	−45899.536	0.218	7.406
Ingroup only, 4 wildcards excluded, Pos3 = ACTG	1	69	ACTG	100M	80M	20M	−77128.707	0.526	−5.712
	2	69	ACTG	100M	80M	20M	−77122.994	0.51	5.712
Ingroup only, 4 wildcards excluded, Pos3 = allRY	1	69	RY	100M	25M	75M	−43366.928	0.28	−6.19
	2	69	RY	100M	25M	75M	−43360.738	0.366	6.19
68 taxa (army ants excluded)	1	68	ACTG	50M	30M	20M	−83784.123	0.301	8.811
	2	68	ACTG	50M	30M	20M	−83792.934	0.283	−8.811

For each treatment (column 1), the two runs of highest harmonic-mean marginal likelihood are reported and compared using Bayes Factors as calculated using 1000 bootstrap pseudoreplicates in Tracer v.1.5, which employs a weighted likelihood bootstrap estimator. Treatments include (i) modifying third positions of protein-coding genes; (ii) excluding four “wildcard” taxa (*Cerapachys splendens, Sphinctomyrmex stali, Tanipone zona,* and *Vicinopone conciliatrix*); (iii) excluding outgroups; (iv) excluding army ants; and (v) selected combinations of the foregoing. Third-position sites of protein-coding genes were modified in order to explore the effects of base frequency heterogeneity across taxa, and consisted of (i) no modifications, i.e., retaining the base identities at third positions (“ACTG”); (ii) coding all third positions as either R (purines, A or G) or Y (pyrimidines, C or T) (“allRY”); (iii) coding as RY the third positions of only a subset of taxa, differing for each gene and depending on the degree of departure of base frequencies from the average, and preserving the base identities of the remainder (“some RY”); and (iv) excluding all third positions (“excluded”).

In order to assess the possibility of conflicting gene trees and to compare gene-specific support for clades, we also conducted analyses of each of the 11 genes separately using the partitioning schemes and models described above and in Table 1. All single-gene analyses were conducted under Bayesian criteria as described above and consisted of 10 million generations with a burnin of 1 million generations. The results of the single-gene analyses are summarized in Additional file 5.

### Maximum likelihood phylogenetic analyses

Partitioned ML analyses were carried out in GARLI-PART 0.97.r737 [66] using parallel processing; most ML bootstrap runs were conducted on the University of Maryland distributed-computing Lattice Project (http://boinc.umiacs.umd.edu/about.php; [67]). ML nucleotide- and codon-model bootstrap analyses consisted of >1000 and >200 pseudoreplicates, respectively (Table 2), and deviated from default settings as follows: genthreshfortopoterm = 5000; scorethreshforterm = 0.10; startoptprec = 0.5; minoptprec = 0.01; numberofprecreductions = 1; treerejectionthreshold = 20.0; topoweight = 0.01; brlenweight = 0.002. ML nucleotide- and codon-model “best tree” analyses consisted of 50 and 10 pseudoreplicates, respectively, and deviated from the default settings as follows: topoweight = 0.01; brlenweight = 0.002. In all analyses the value for modweight was calculated as 0.0005 × (#subsets + 1) (D. Zwickl, personal communication).

### Divergence date estimation

We inferred divergence dates for Dorylinae lineages using BEAST v1.7.5 [68] under a parallel configuration on BEAGLE v1.0. We employed an uncorrelated lognormal relaxed clock model [69]. Data partitioning and model selection were the same as for the MrBayes analyses described above. Substitution models were unlinked and clock and tree models were linked among partitions. The tree prior was a Yule process with a random starting topology. We provided *a priori* age distributions for six stem group nodes based on the fossil record (Table 3). The relevant fossils for each calibrated node are: A, *Acanthostichus* and *Cylindromyrmex* in Dominican amber [31,70,71]; B, *Neivamyrmex* in Dominican amber [72]; C, two species of *Procerapachys* in Baltic and Bitterfeld ambers [73,74], conservatively used to calibrate the stem group inclusive of all *Cerapachys* species; D, *Prionomyrmex* in Baltic amber [75,76]; E, Myrmeciinae in Olst Formation, Denmark [77]; F, diverse Dolichoderinae in Sakhalin amber (Paleocene) and possibly in Medicine Hat amber [78]. An initial BEAST analysis excluding the data matrix was conducted to verify that our inferred posterior age distributions were not driven solely by the actual marginal prior distributions resulting from multiplicative construction [79]. Stationarity and burnin were determined by observing high (>200) ESS values and the consistency of results among independent runs. We conducted BEAST runs for 200 million generations with a burnin of 40 million generations.

**Table 3:** **Lognormal *a priori *** **age distribution parameters (in millions of years) used for node calibrations, applied to stem group taxa based on evidence from the fossil record**

**Taxon**	**Offset**	**Median ** **age**	**95% quantile**	**Log (mean)**	**Log (s.d.)**
*Acanthostichus* and *Cylindromyrmex*	15	50	70	3.55	0.28
*Neivamyrmex*	15	40	70	3.22	0.48
*Procerapachys*	42	80	100	3.64	0.26
*Prionomyrmex*	42	60	80	2.90	0.45
Myrmeciinae	54.5	95	110	3.70	0.20
Dolichoderinae	60	95	120	3.56	0.33

## Availability of supporting data

The data matrix supporting the results of this article is available in Additional file 6. This data matrix and all phylogenetic tree files are also available from the Dryad Digital Repository: http://dx.doi.org/10.5061/dryad.3df32[80]. New sequence data generated for this study have been deposited in GenBank (http://www.ncbi.nlm.nih.gov/genbank/) under accession numbers KJ523183-KJ523884. GenBank accession numbers for all sequences used in this study are listed in Additional file 3.

## Competing interests

The authors declare that they have no competing interests.

## Authors’ contributions

SGB, BLF, TRS, and PSW conceived the study and participated in its design and coordination. SGB generated and edited molecular data, conducted divergence dating analyses, and led manuscript writing. BLF obtained specimens, databased specimen information and images, and helped to draft the manuscript. TRS conducted phylogenetic analyses and helped to draft the manuscript. PSW obtained specimens, databased specimen information, generated and edited molecular data, and helped to draft the manuscript. All authors read and approved the final manuscript.

## Supplementary Material

Additional file 1 Table of support values for select groups under different analytical treatments. Click here for file

Additional file 2 Phylogenetic reconstructions from all data treatments. Click here for file

Additional file 3 **Taxa included in the study with GenBank accession numbers and specimen voucher information.** Detailed collection data for each species is available by searching for the specimen code on AntWeb (http://www.antweb.org). Click here for file

Additional file 4 Summary of rate heterogeneity values for third-position partitions of protein-coding genes, which informed the partial RY coding analyses. Click here for file

Additional file 5 Support values for select groups from Bayesian analyses of individual genes. Click here for file

Additional file 6 Full phylogenetic data matrix, including definitions of character partitions and exclusion sets. Click here for file
