# Supplementary material for: The rise of army ants and their relatives: diversification of specialized predatory doryline ants
Source: BMC Evol Biol. 2014 May 1;14:93. doi: 10.1186/1471-2148-14-93 (PMC4021219; doi:10.1186/1471-2148-14-93)

Phylogenetic reconstructions from all data treatments. See the Methods section in the paper for details about the different data treatments. Trees are presented in the same order as found in Table 2, with single gene trees added to the end of this file. The order of trees in the file is given below. MB = MrBayes; ML = maximum likelihood.

Page	Treatment
3	83 taxa (all), ACTG coding, MB
4	83 taxa (all), ACTG coding, ML bootstraps
5	83 taxa (all), ACTG coding, ML best tree
6	83 taxa (all), full RY coding, MB
7	83 taxa (all), full RY coding, ML bootstraps
8	83 taxa (all), partial RY coding, MB
9	83 taxa (all), partial RY coding, ML bootstraps
10	83 taxa (all), third codon positions excluded, MB
11	83 taxa (all), third codon positions excluded, ML bootstraps
12	83 taxa (all), ACTG coding, BEAST
13	83 taxa (all), codon model, ML bootstraps
14	83 taxa (all), codon model, ML best tree
15	79 taxa (excluding 4 wildcard taxa), ACTG coding, MB
16	79 taxa (excluding 4 wildcard taxa), ACTG coding, ML bootstraps
17	79 taxa (excluding 4 wildcard taxa), full RY coding, MB
18	79 taxa (excluding 4 wildcard taxa), full RY coding, ML bootstraps
19	79 taxa (excluding 4 wildcard taxa), partial RY coding, MB
20	79 taxa (excluding 4 wildcard taxa), partial RY coding, ML bootstraps
21	79 taxa (excluding 4 wildcard taxa), third codon positions excluded, MB
22	79 taxa (excluding 4 wildcard taxa), third codon positions excluded, ML bootstraps
23	79 taxa (excluding 4 wildcard taxa), codon model, ML bootstraps
24	73 taxa (ingroup only), ACTG coding, MB
25	73 taxa (ingroup only), full RY coding, MB
26	69 taxa (ingroup only and excluding 4 wildcard taxa), ACTG coding, MB
27	69 taxa (ingroup only and excluding 4 wildcard taxa), full RY coding, MB
28	68 taxa (excluding army ants), ACTG coding, MB
29	83 taxa (all), ACTG coding, 18S only, MB
30	83 taxa (all), ACTG coding, 28S only, MB
31	83 taxa (all), ACTG coding, abdA only, MB
32	83 taxa (all), ACTG coding, argK only, MB
33	83 taxa (all), ACTG coding, CAD only, MB
34	83 taxa (all), ACTG coding, EF1aF1 only, MB

35 83 taxa (all), ACTG coding, EF1aF2 only, MB
36 83 taxa (all), ACTG coding, LWRh only, MB
37 83 taxa (all), ACTG coding, Top1 only, MB
38 83 taxa (all), ACTG coding, Ubx only, MB
39 83 taxa (all), ACTG coding, wg only, MB

Dory83, Bayesian nuc models, Pos3s included, 100M gens, burnin=50M

Majority-rule consensus tree

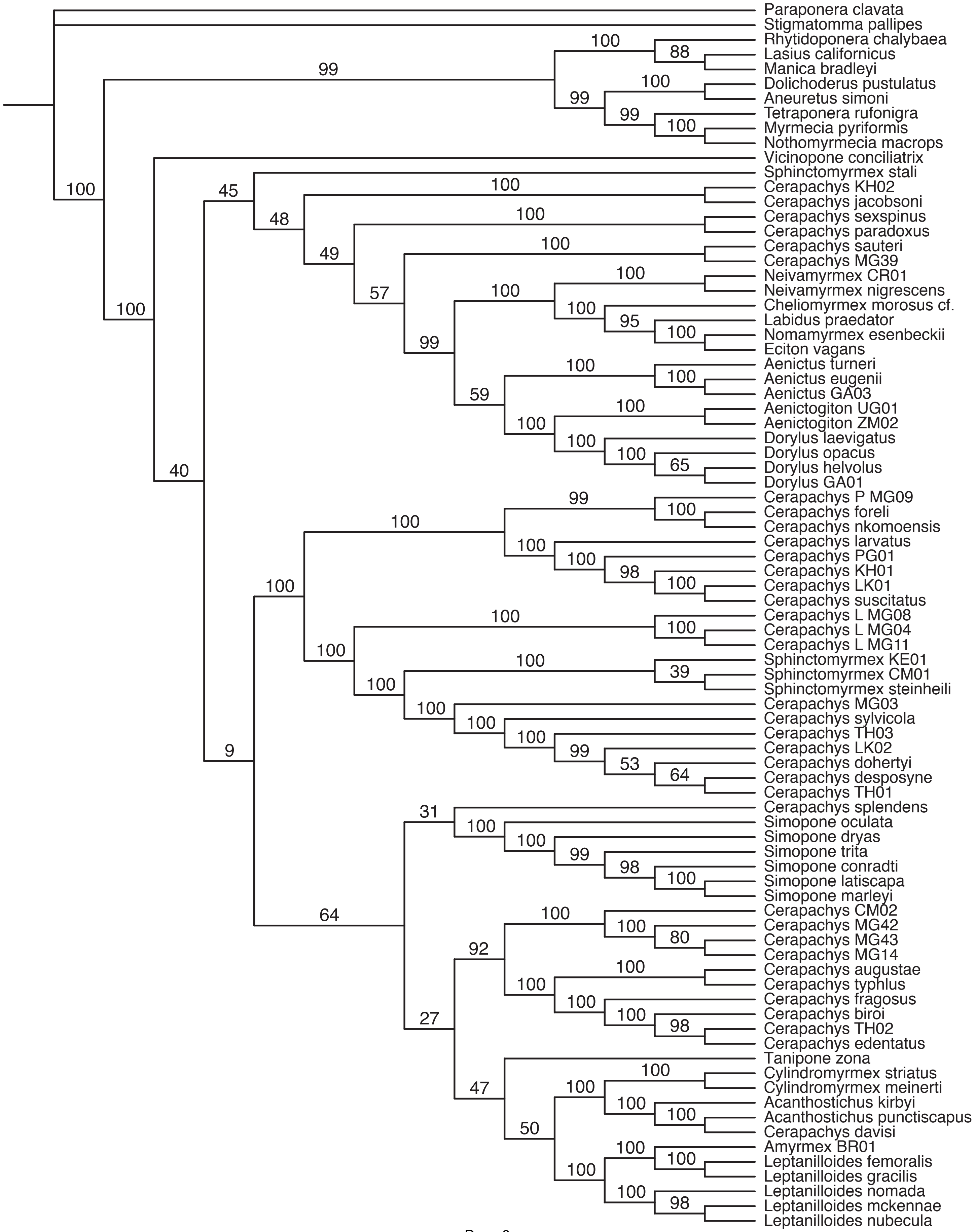

Dory83, pos3=ACTG, partitioned MLBS, 1075 pseudoreps

Majority-rule consensus tree

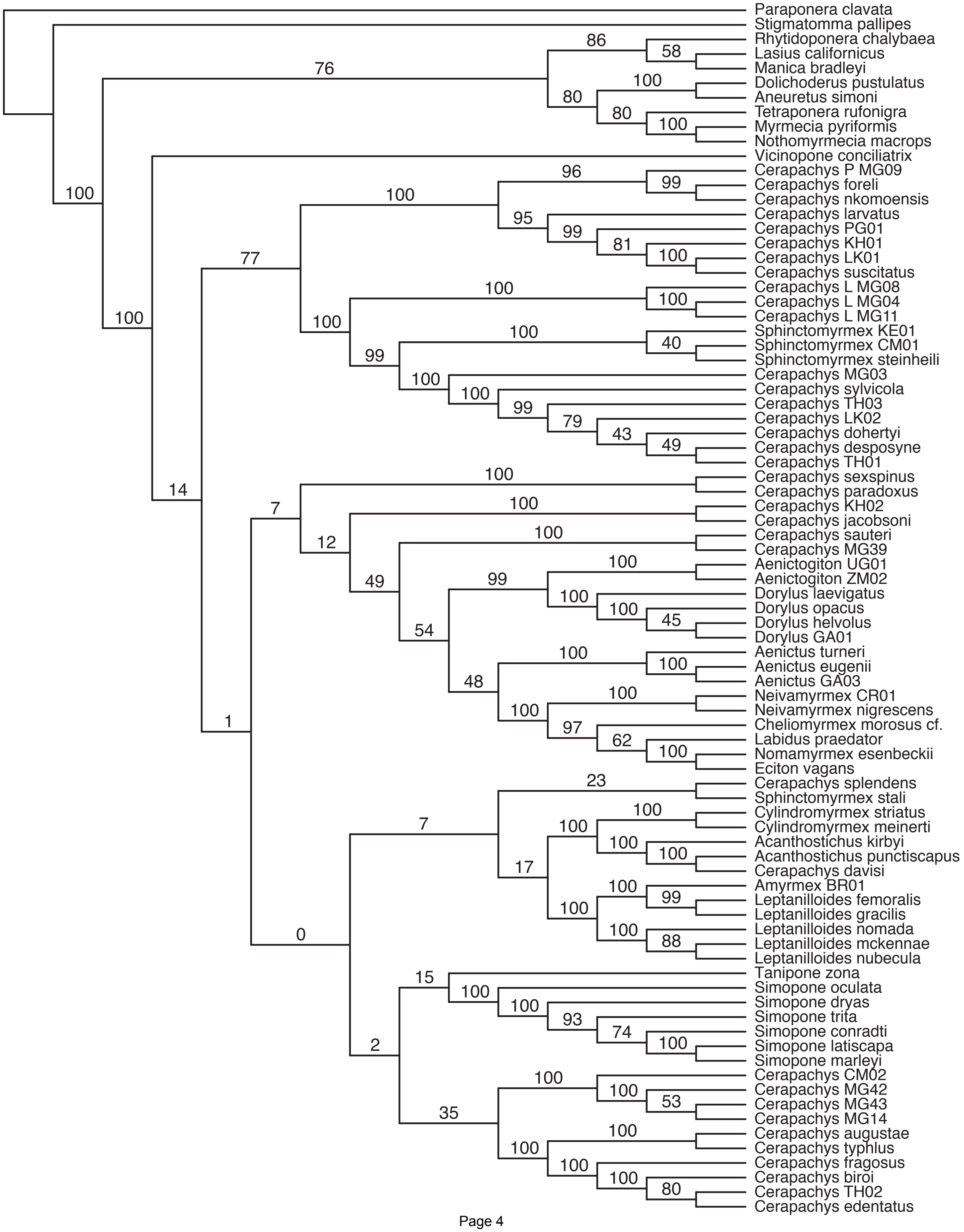

Dorylomorph 83 taxa, 25 partitions, Garli ML Best Tree of 50 searches, lnL=-100757.836560

bestREP4

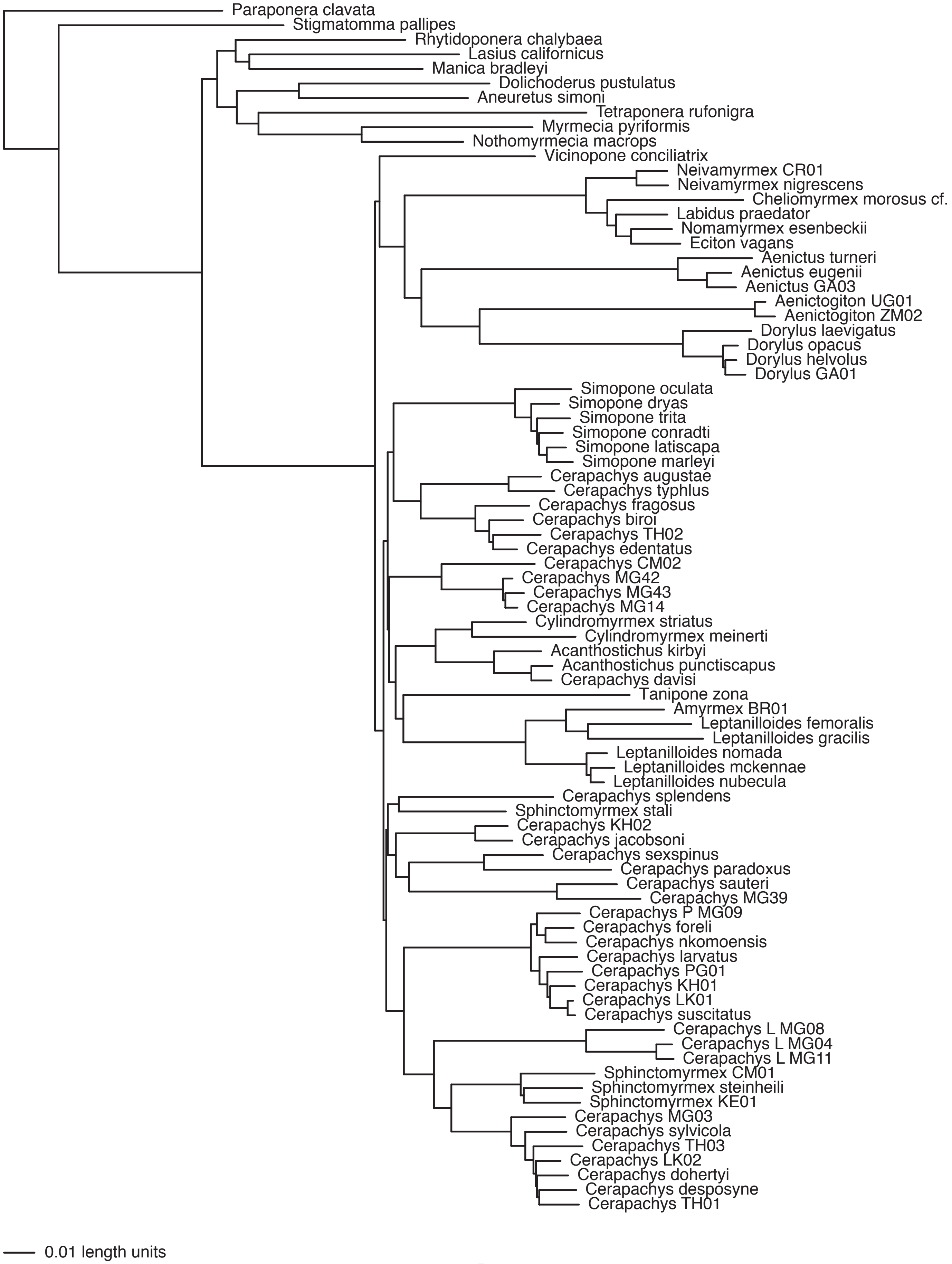

Dory83, Bayesian nuc models, all 3rds coded as RY, 100M gens, burnin=10M

Majority-rule consensus tree

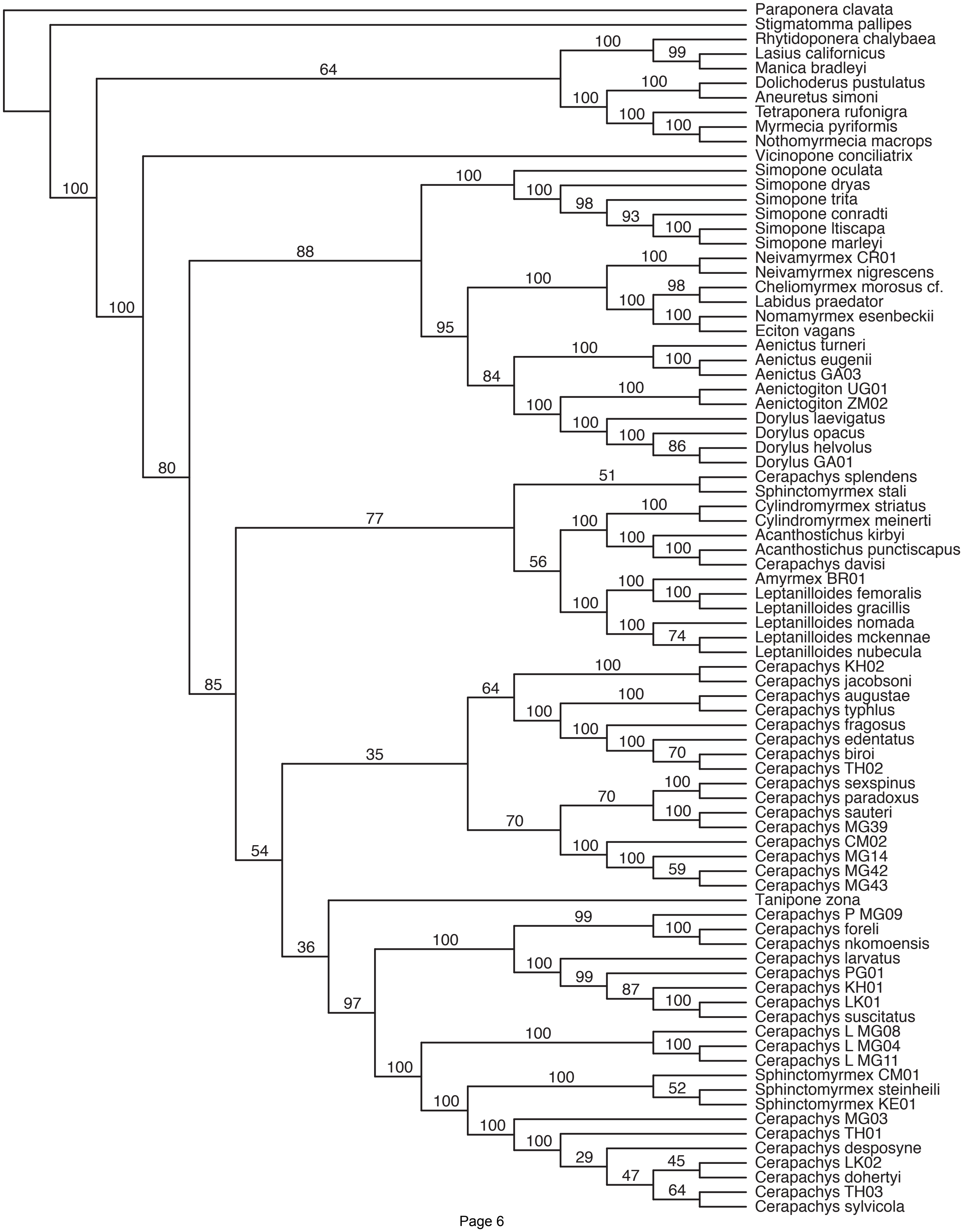

Dory83, pos3s all RY coded, partitioned MLBS, 1090 pseudoreps

Majority-rule consensus tree

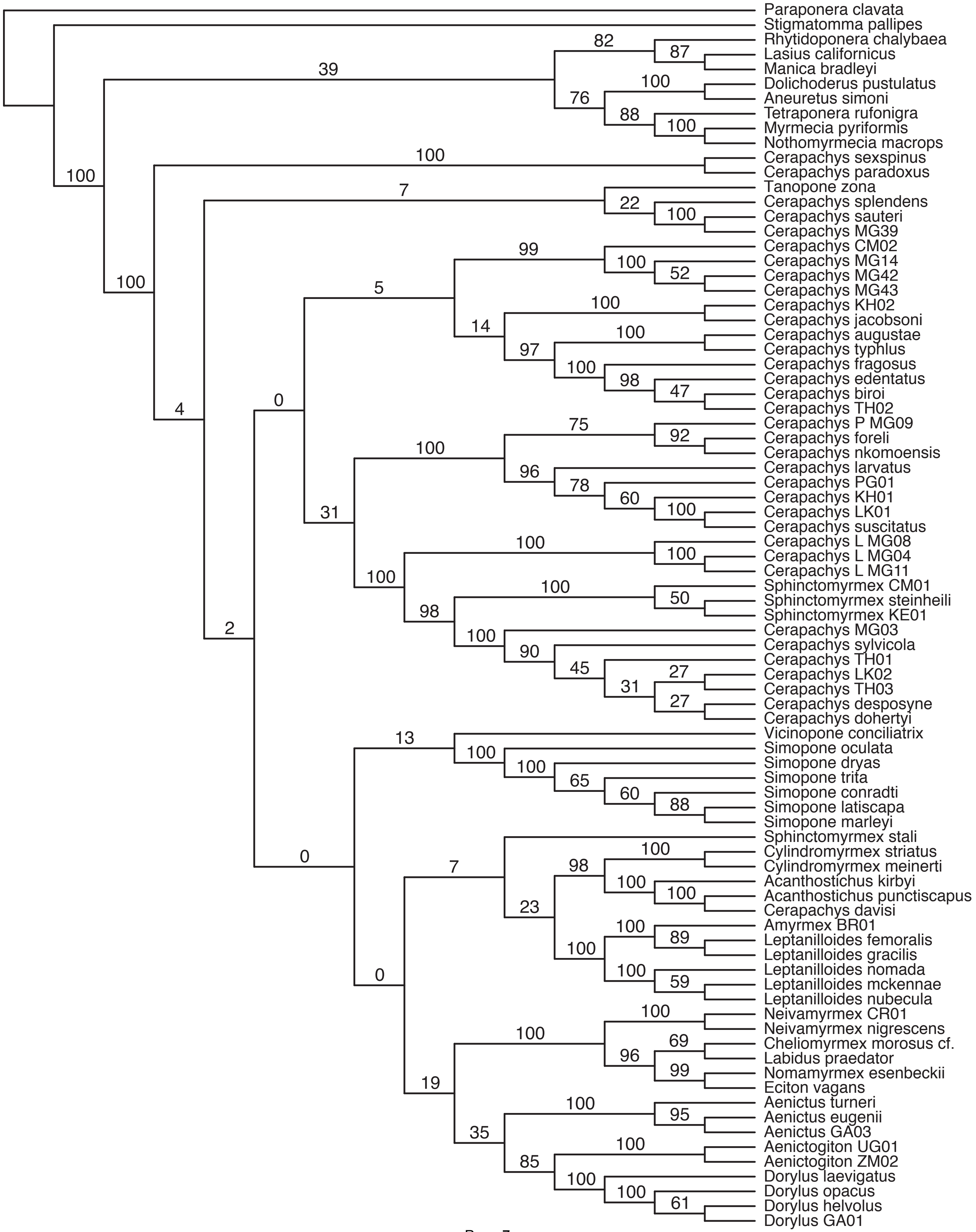

Dory83, subset of pos3s coded as RY, Bayesian, 100M gens, burnin=50M

Majority-rule consensus tree

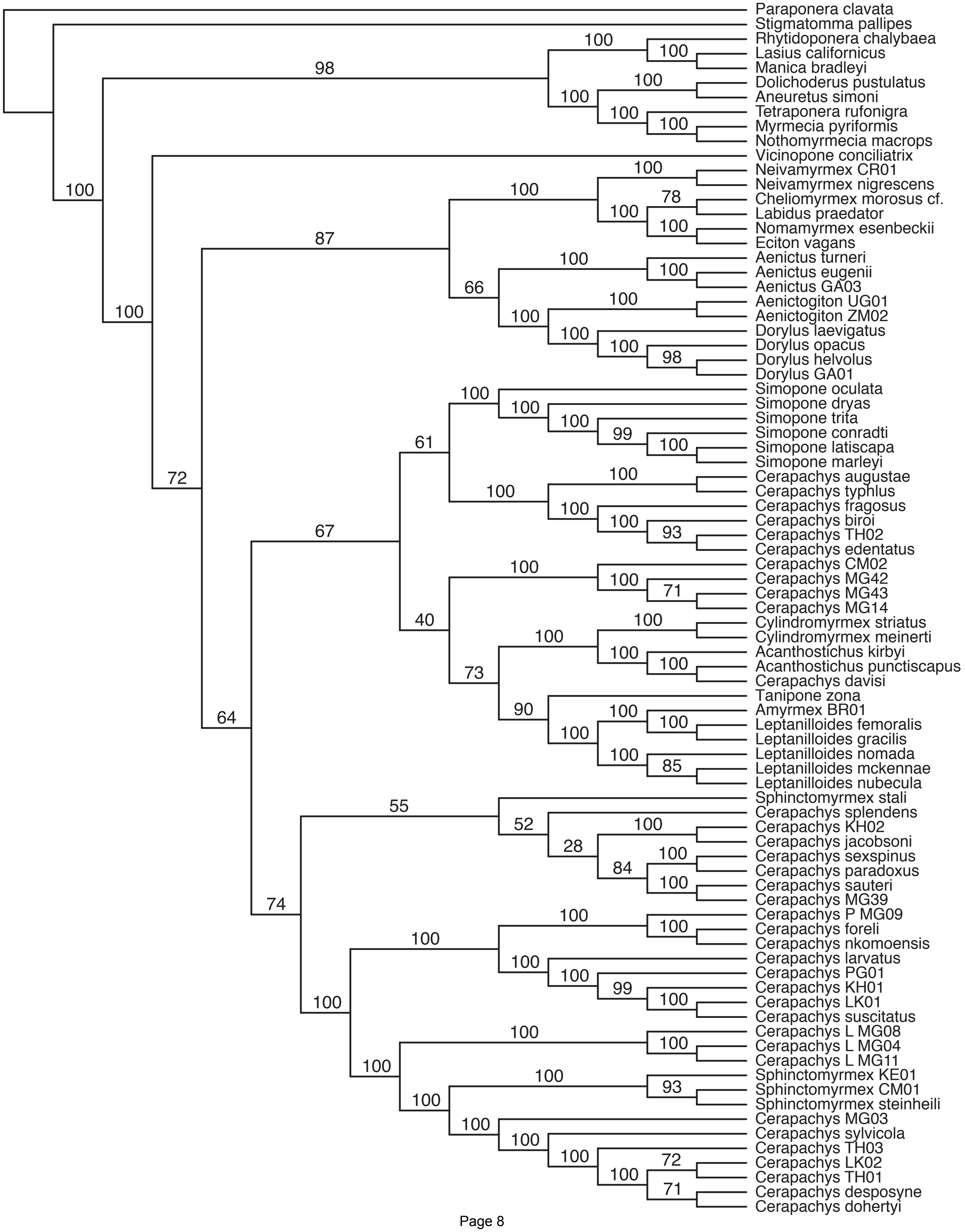

Dory83, subset of pos3s RY coded, partitioned MLBS, 1070 pseudoreps

Majority-rule consensus tree

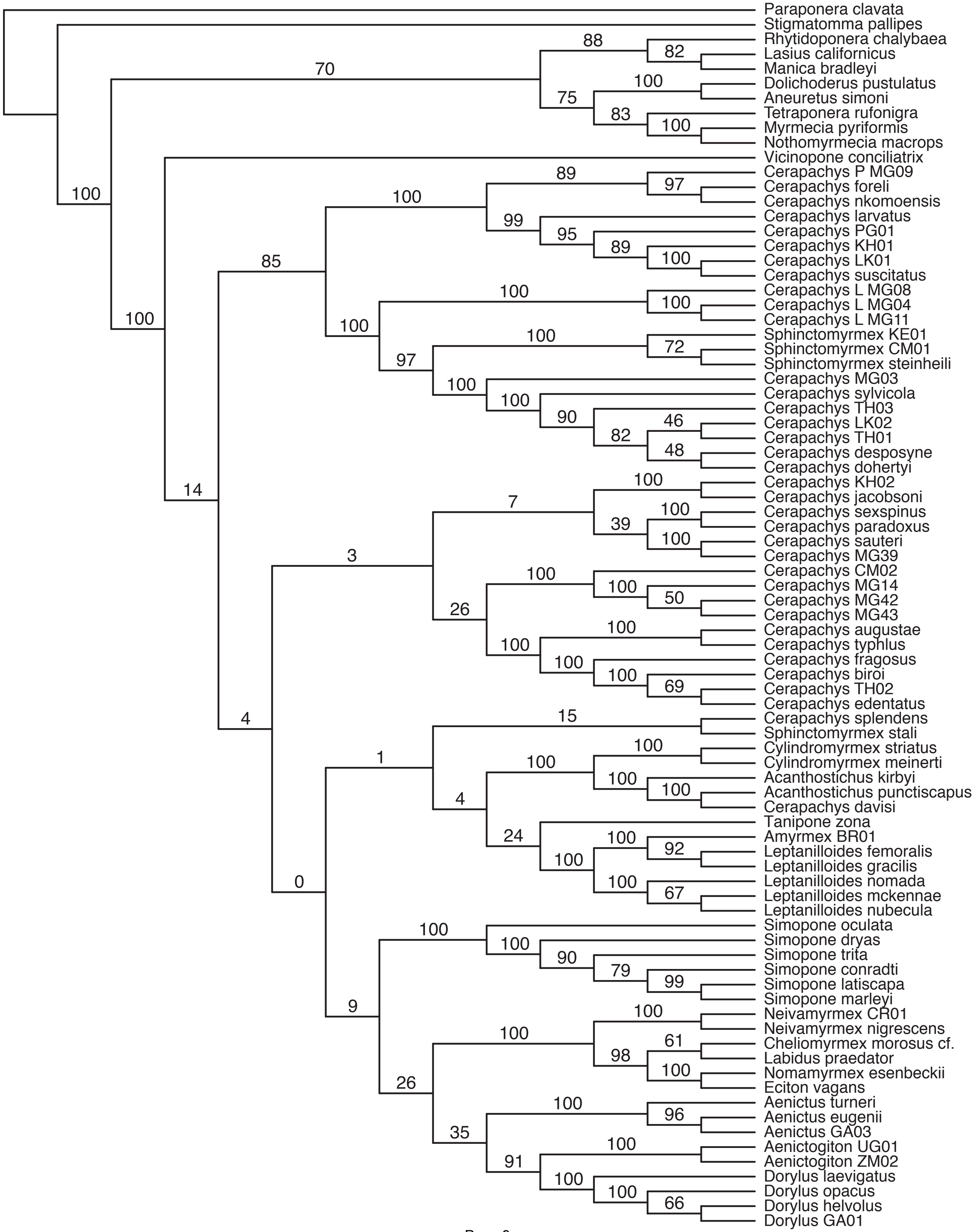

Dory83, MB nuc partitioned model, pos3s excluded, 50M gens, burnin=5M

Majority-rule consensus tree

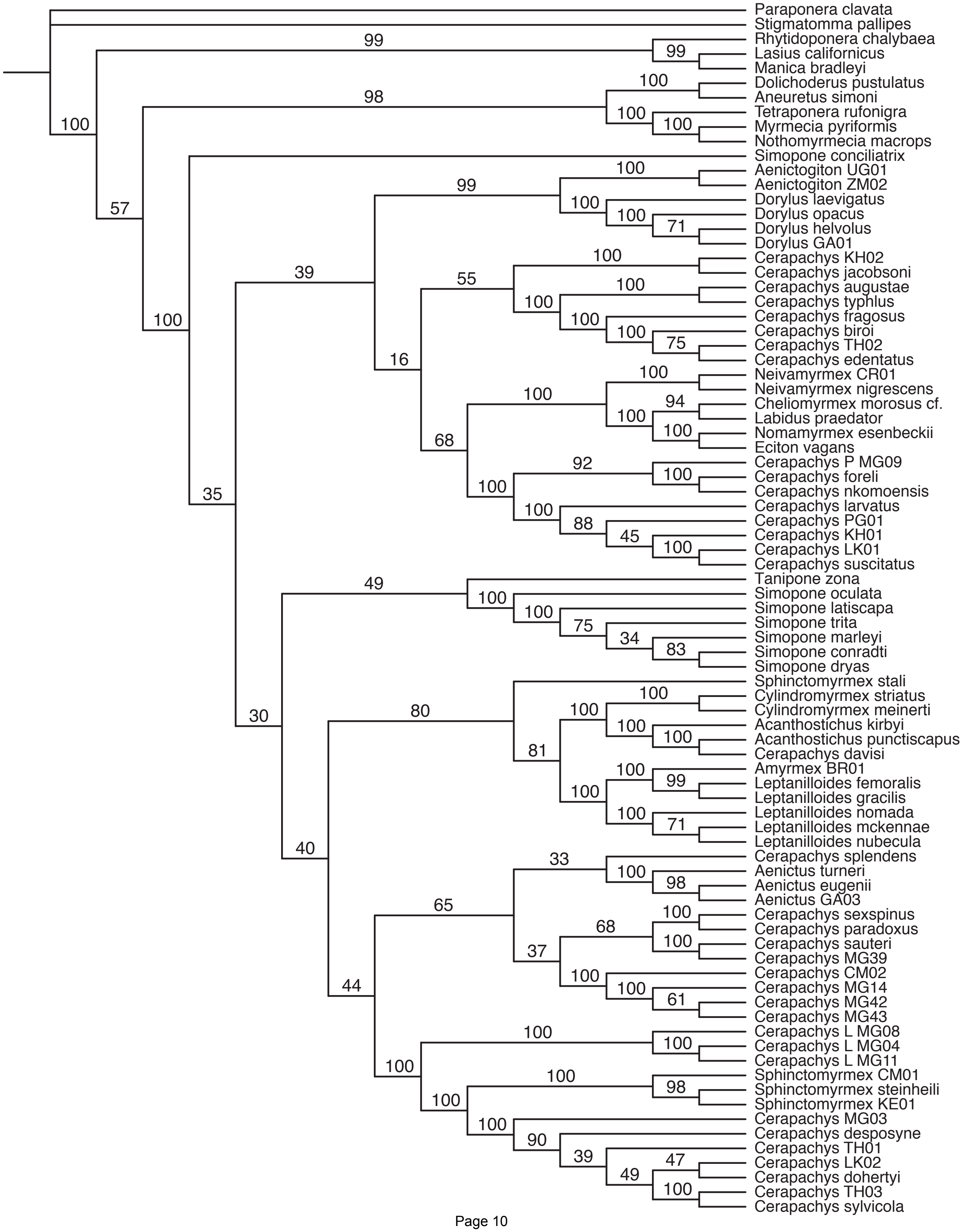

Dory83, 3rd positions excluded, MLBS 1190 pseudoreps

Majority-rule consensus tree

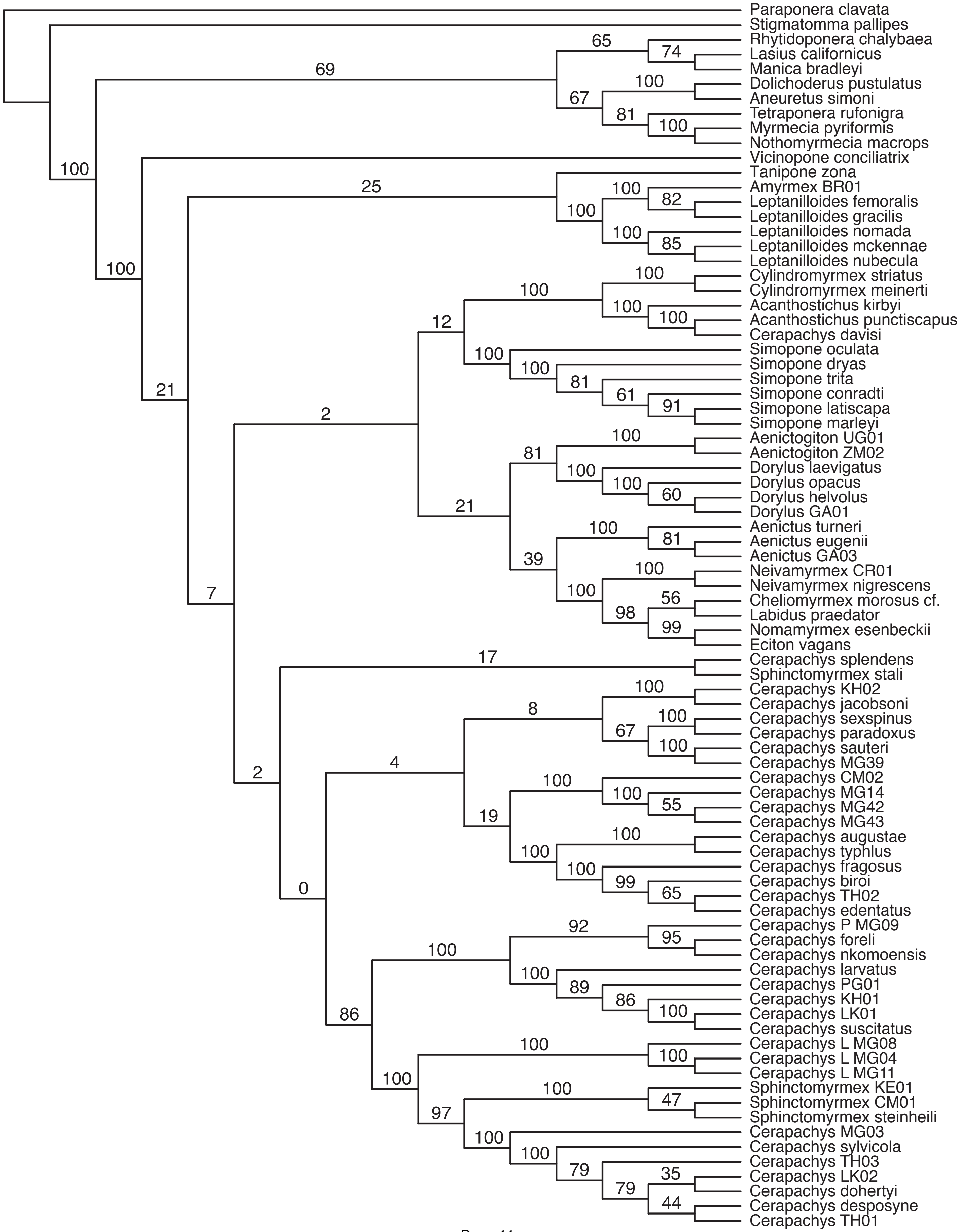

Dory83 (all taxa), ATGC nuc coding, BEAST

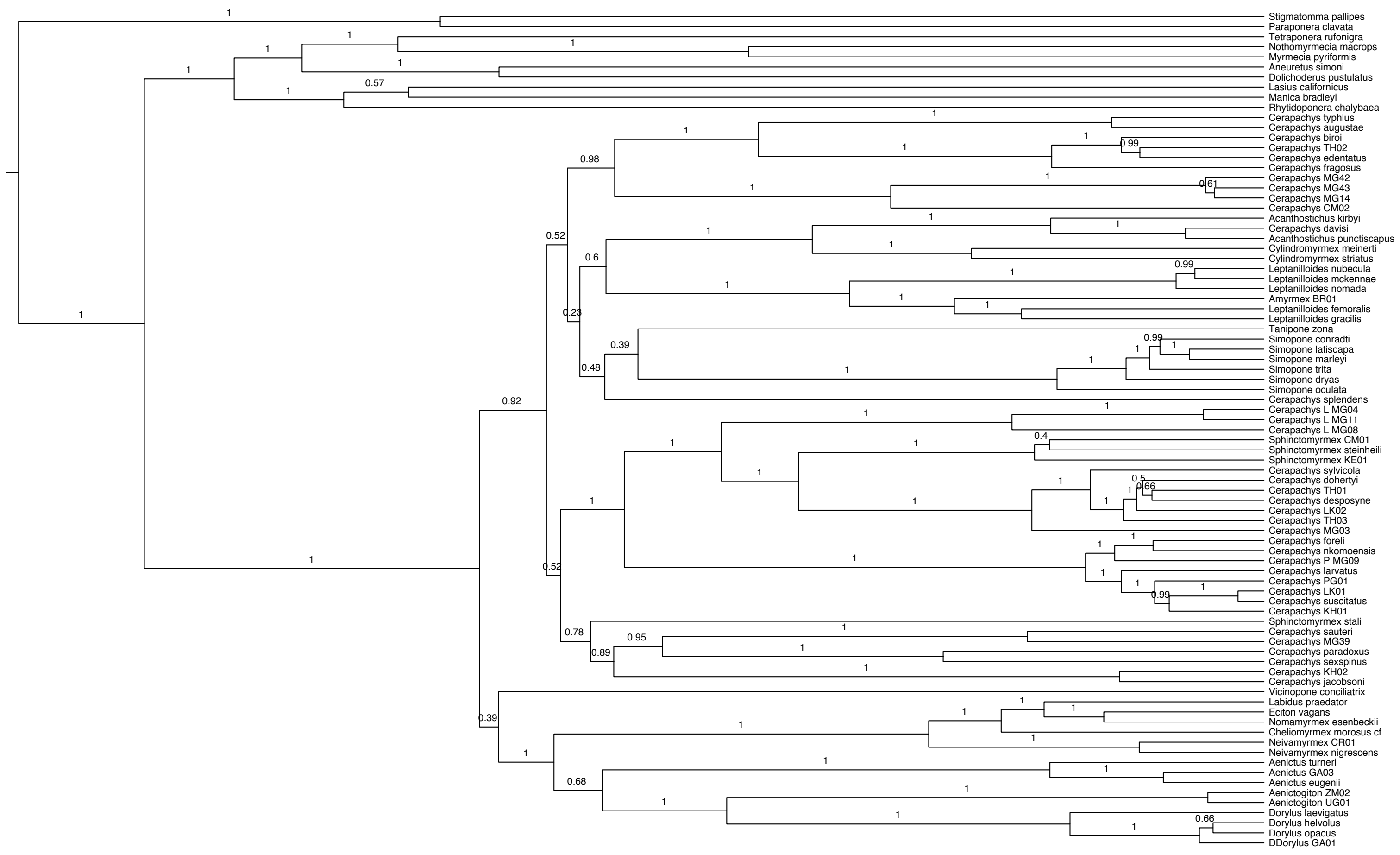

Dory83 partitioned codon model MLBS 228 pseudoreps

Majority-rule consensus tree

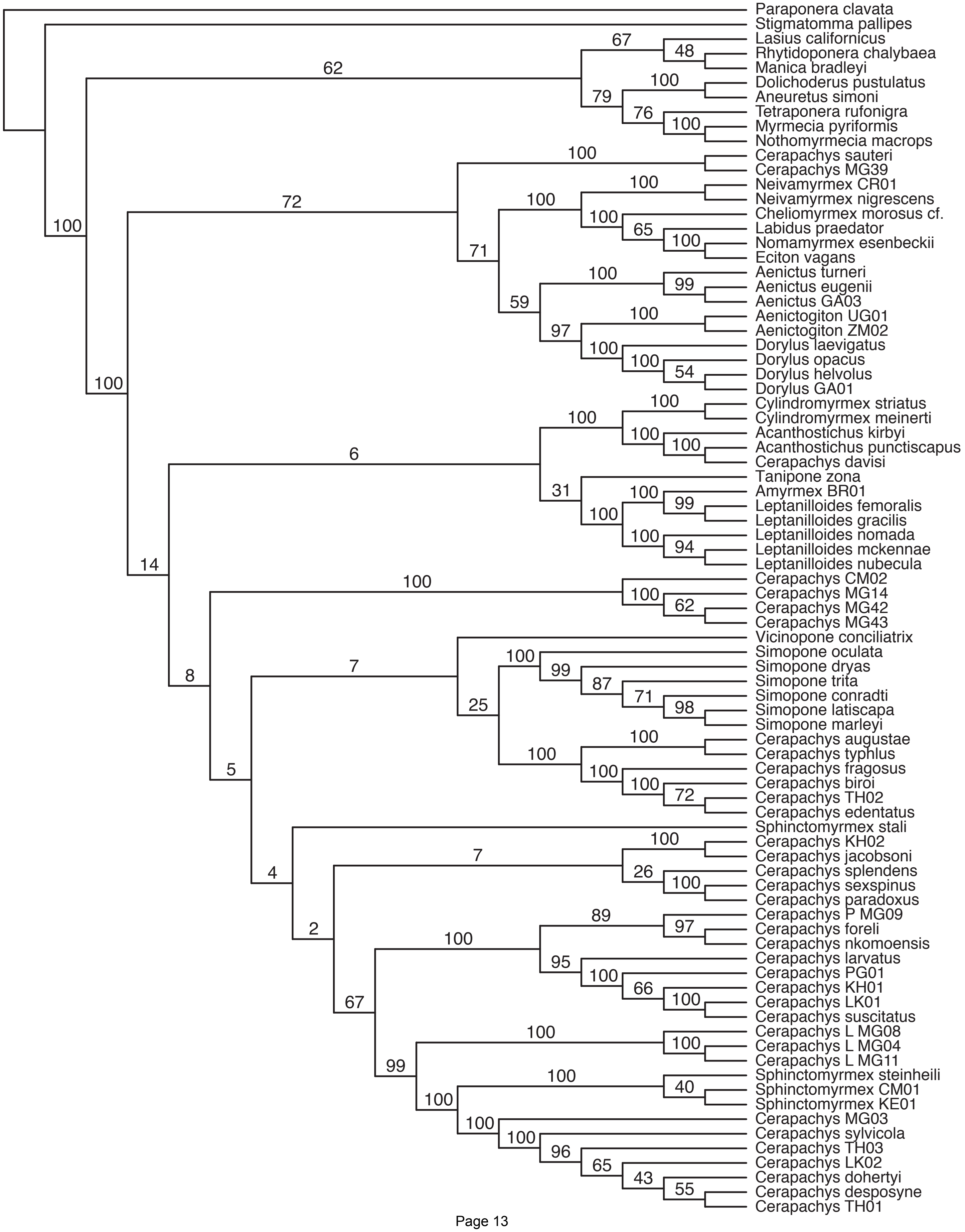

ML codon model, 10 partitions (by gene), best of 10 searches, lnL=-101618.592264

bestREP2

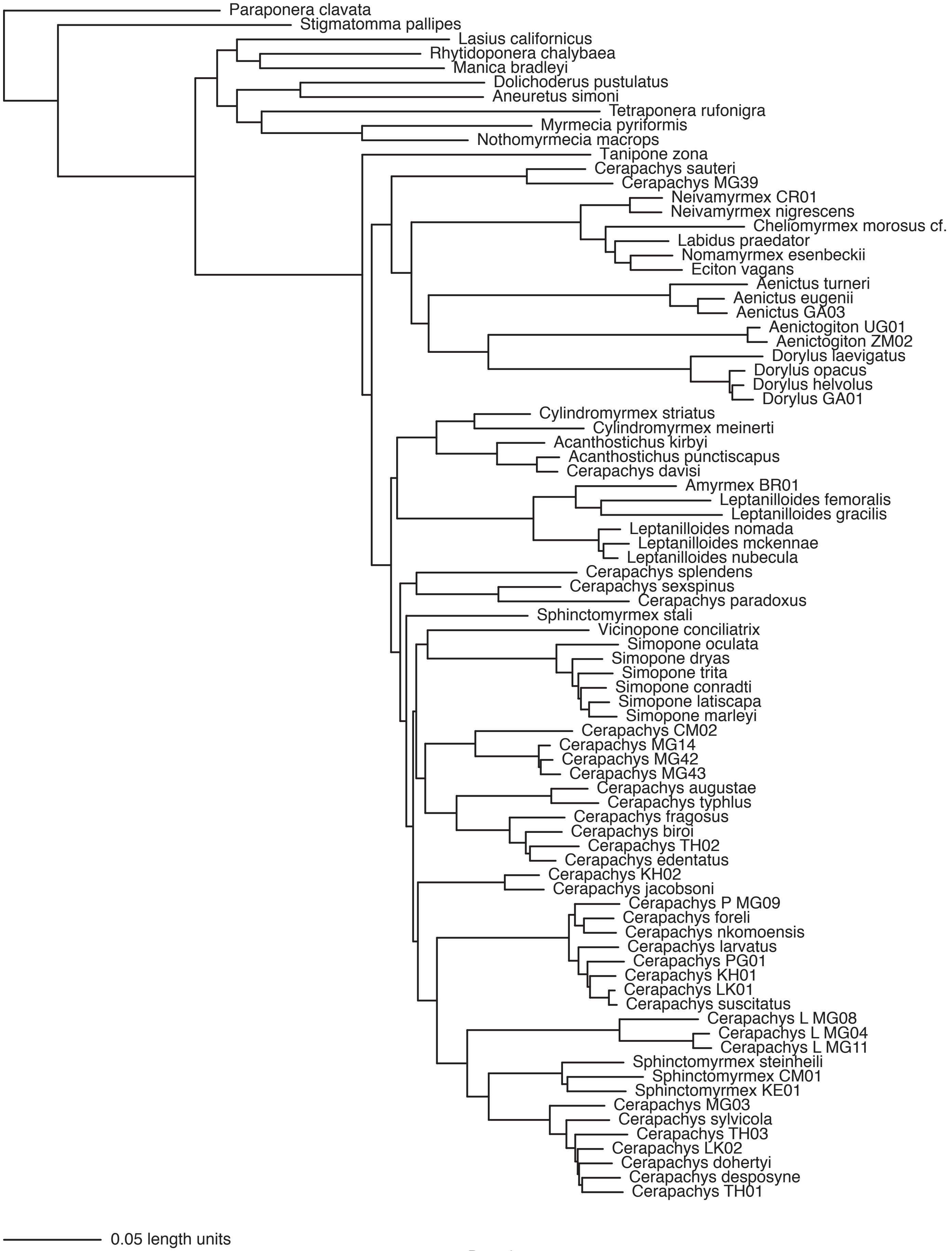

Dory79, 3rd positions coded ACTG, Bayesian, 100M gens, Run1 burnin=20M, Run2 burnin=80M

Majority-rule consensus tree

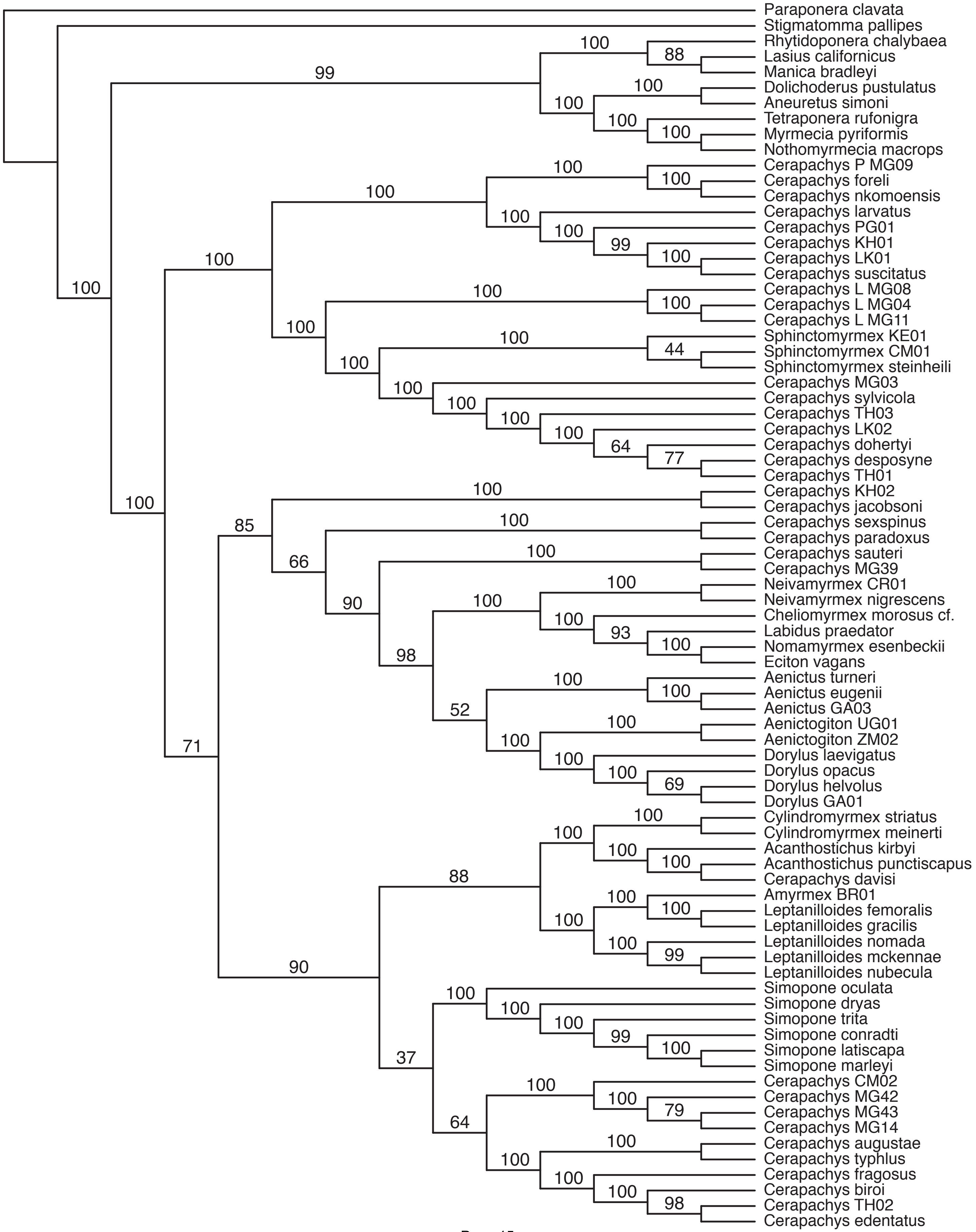

Dory79 (4 wildcard taxa excluded), 3rd positions coded normally (ACTG), MLBS, 1280 pseudoreps

Majority-rule consensus tree

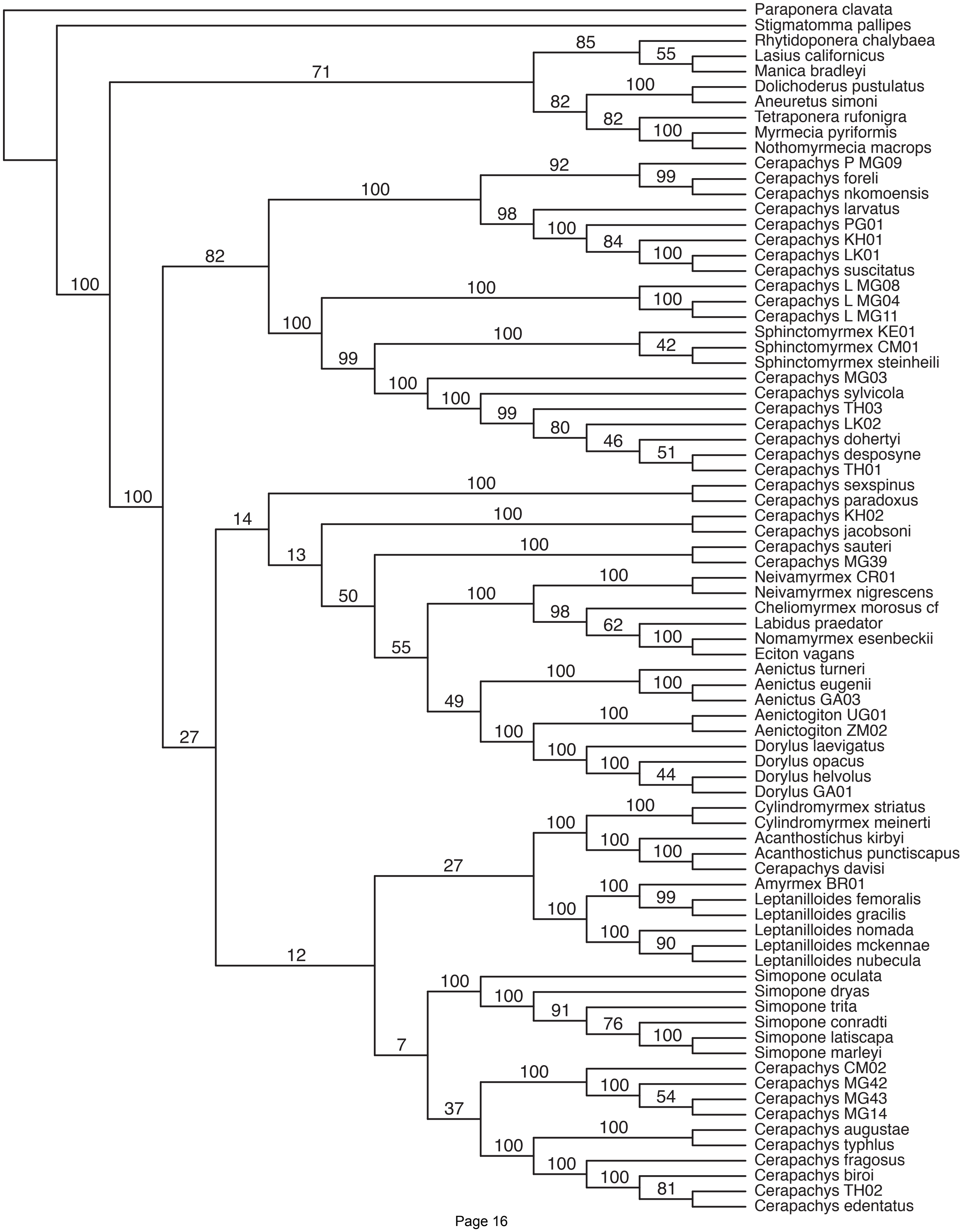

Dory79, all pos3s coded RY, Bayesian, 100M gens, burnin=60M

Majority-rule consensus tree

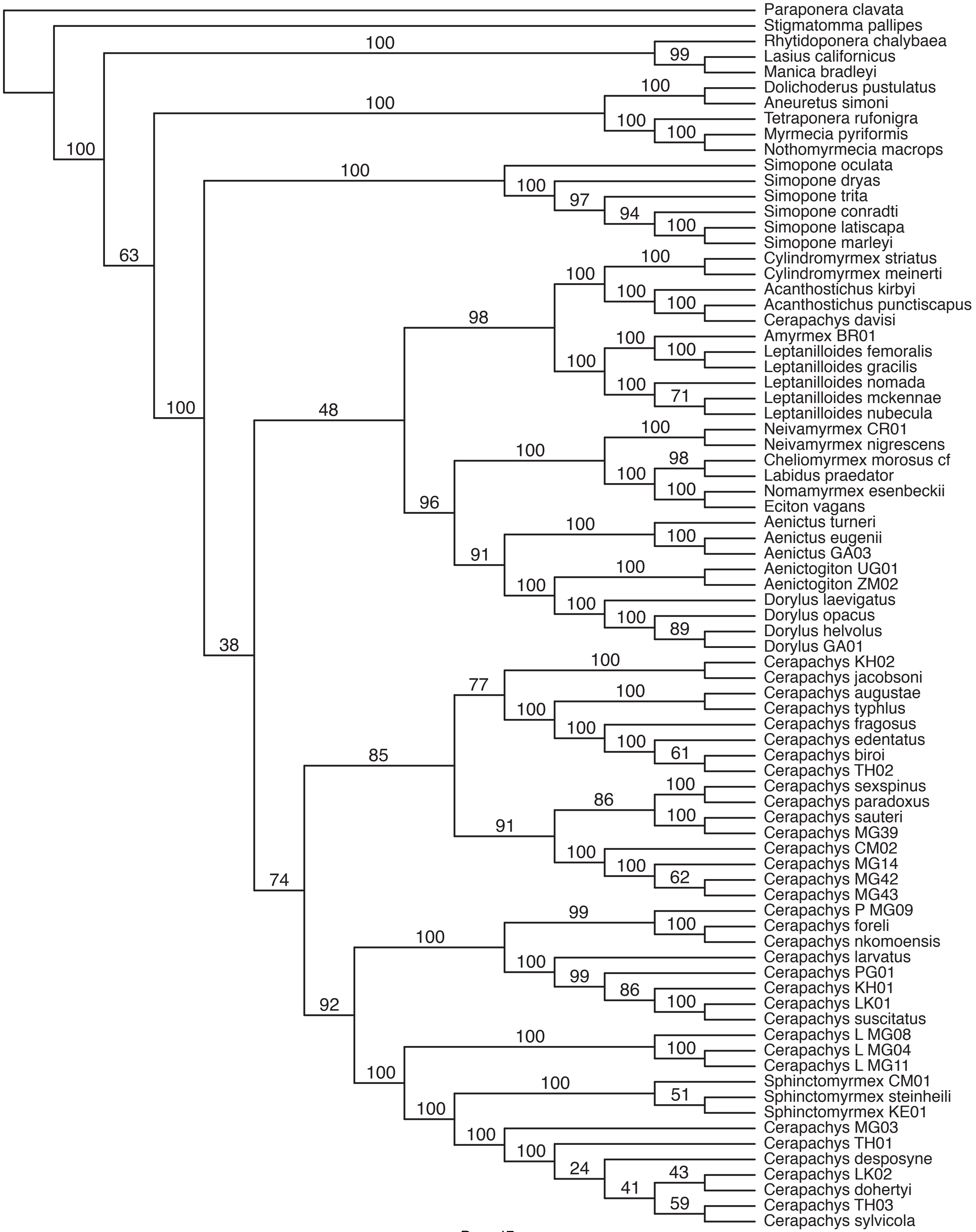

Dory79 (4 wildcard taxa excluded), all 3rd positions coded RY, MLBS, 1022 pseudoreps

Majority-rule consensus tree

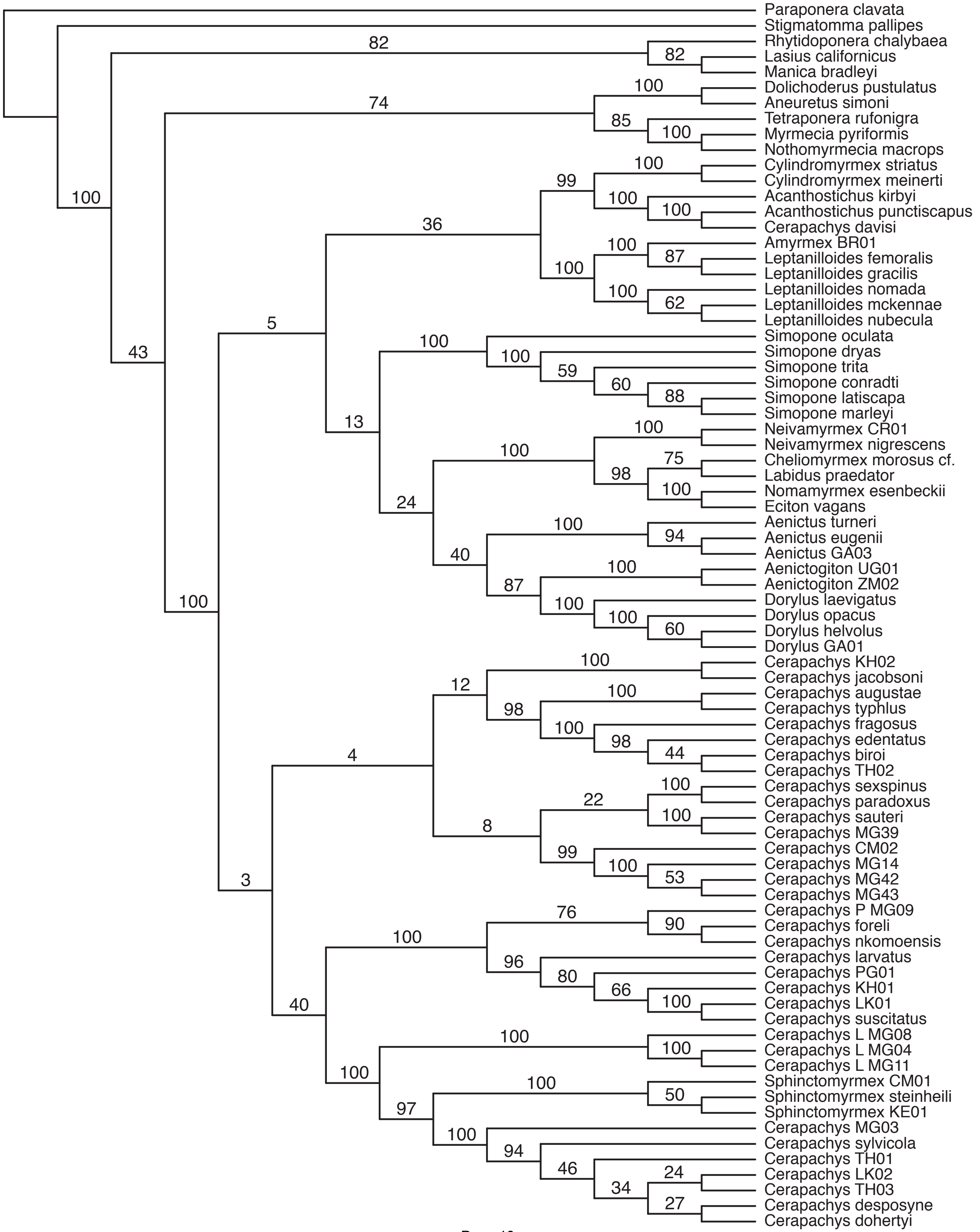

Dory79, subset of 3rds coded RY, Bayesian 100M gens, Run2sr2 bi=50M, best of 4 runs, stationarity, others did not converge

Majority-rule consensus tree

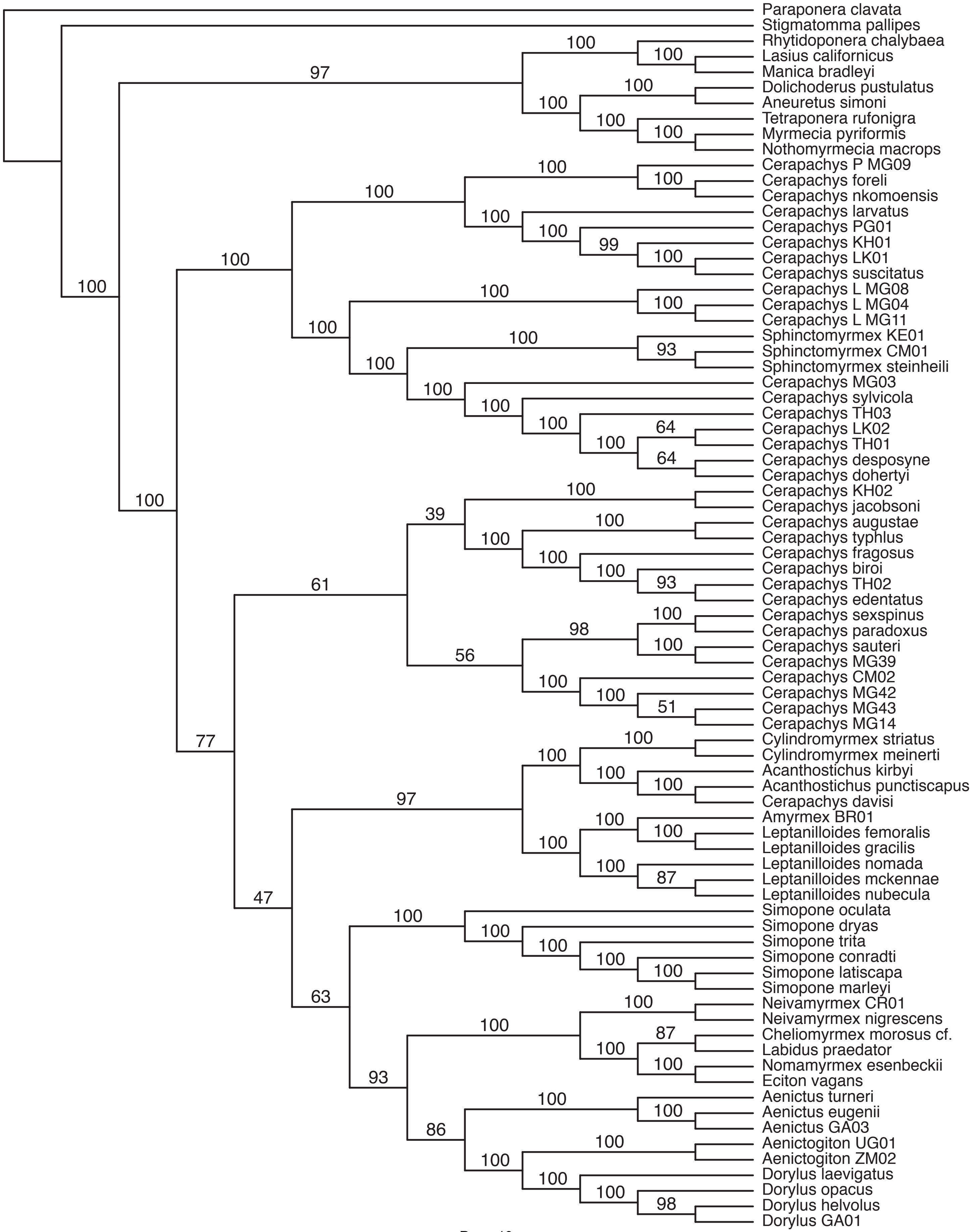

Dory79, subset of pos3s RY coded, partitioned MLBS, 1070 pseudoreps

Majority-rule consensus tree

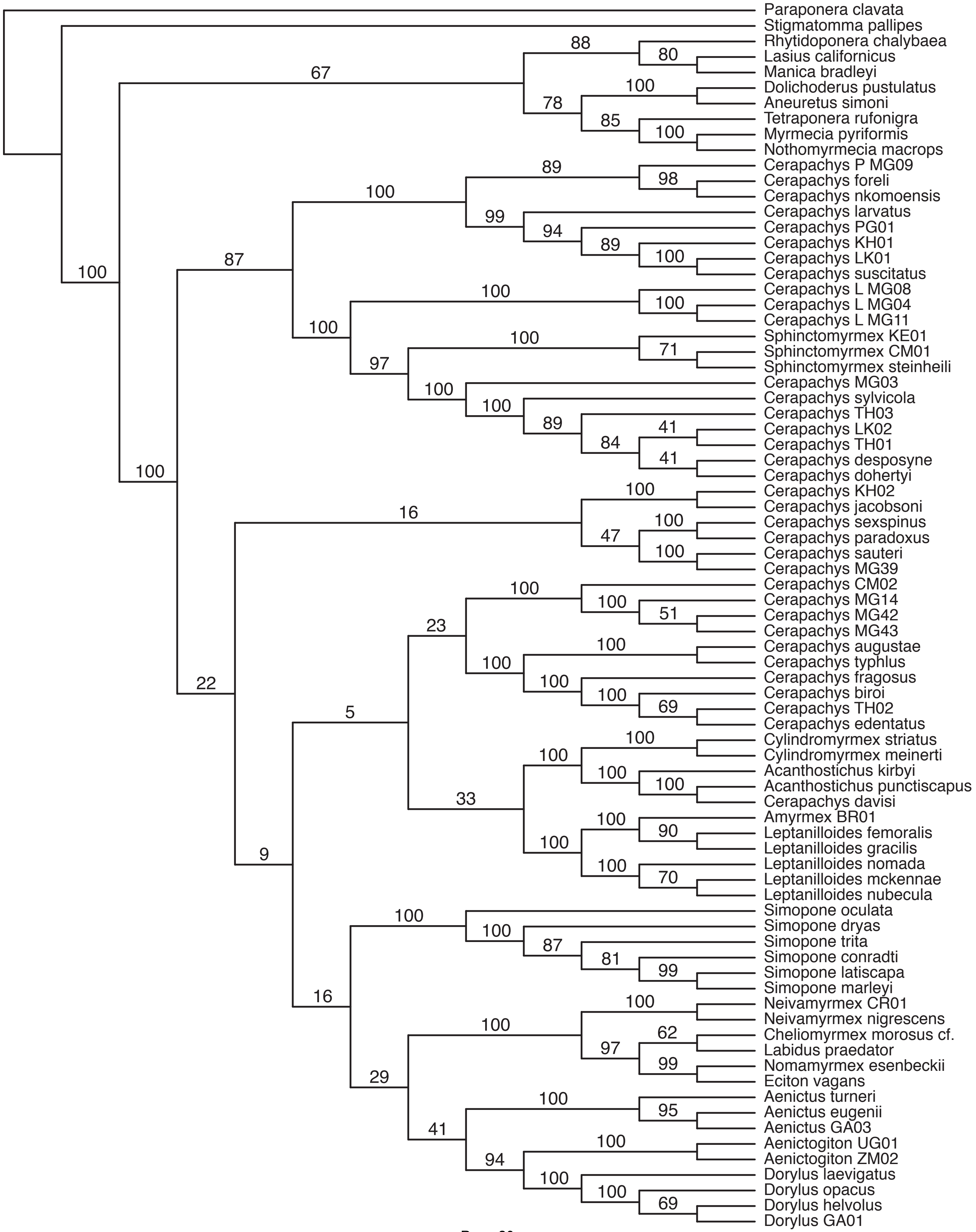

Dory79 (4 wildcard taxa excluded), Bayesian, 50M, burnin=5M, clear stationarity

Majority-rule consensus tree

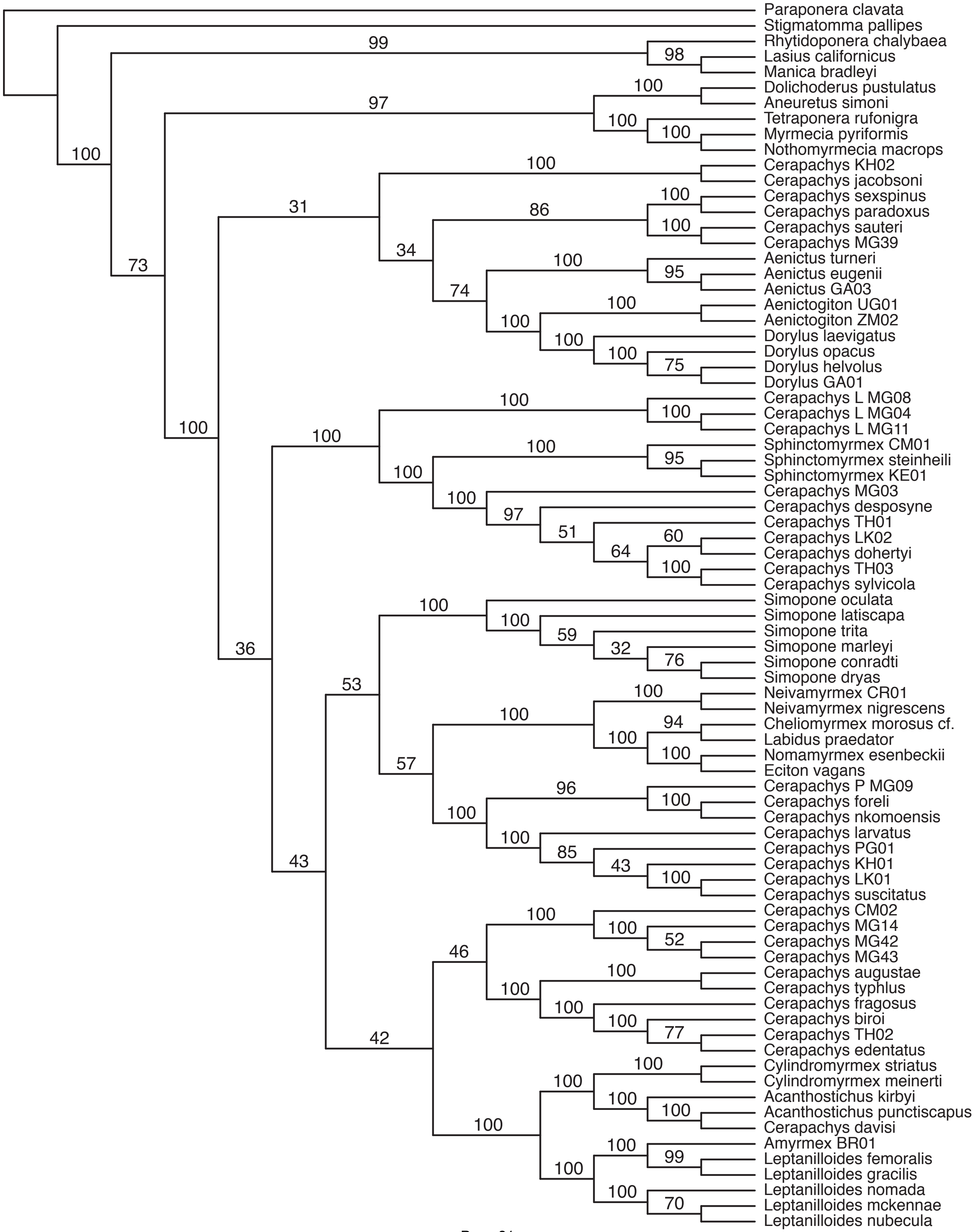

Dory79, pos3s excluded, partitioned MLBS, 1070 pseudoreps

Majority-rule consensus tree

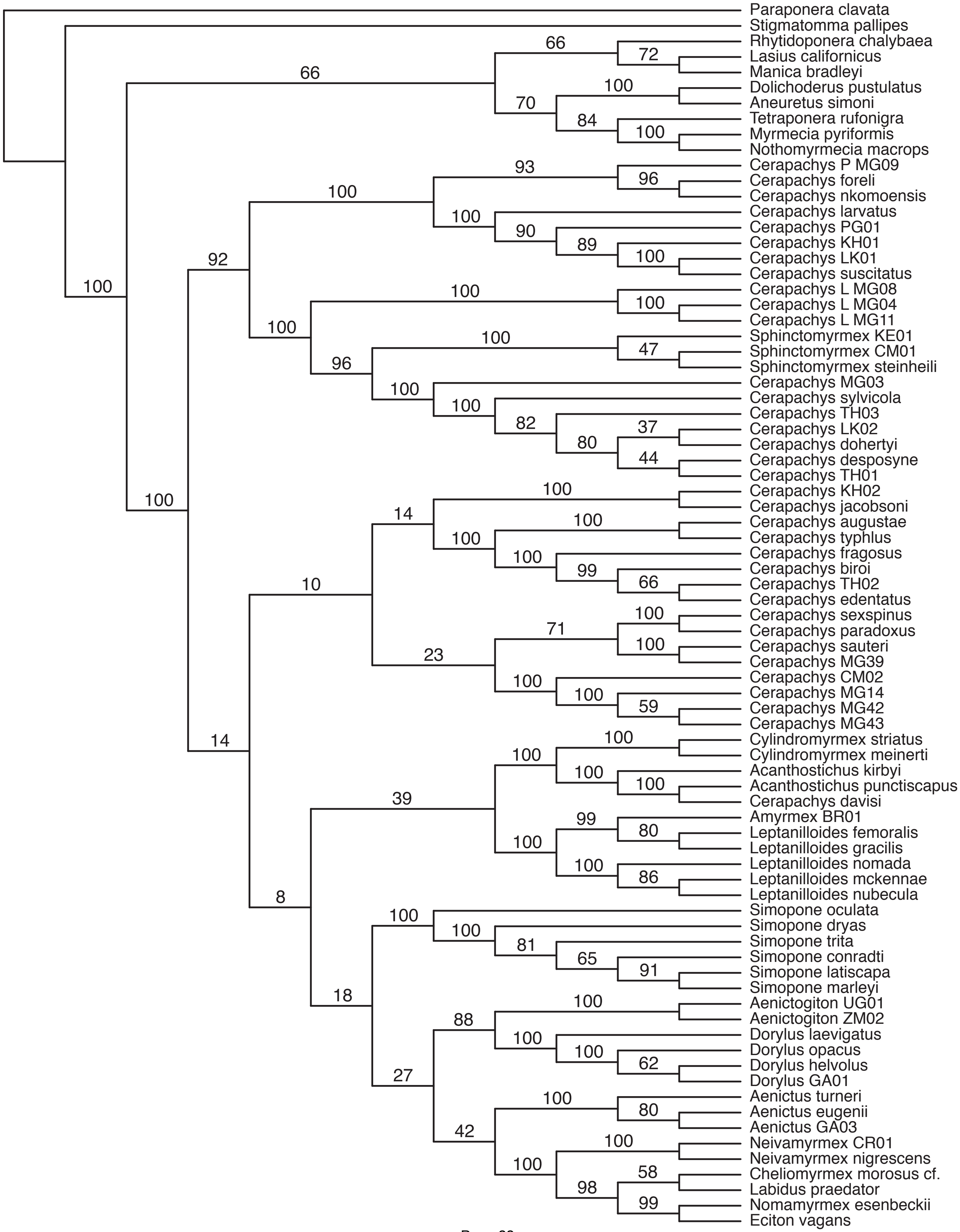

Dory79, codon model, MLBS, 209 pseudoreps

Majority-rule consensus tree

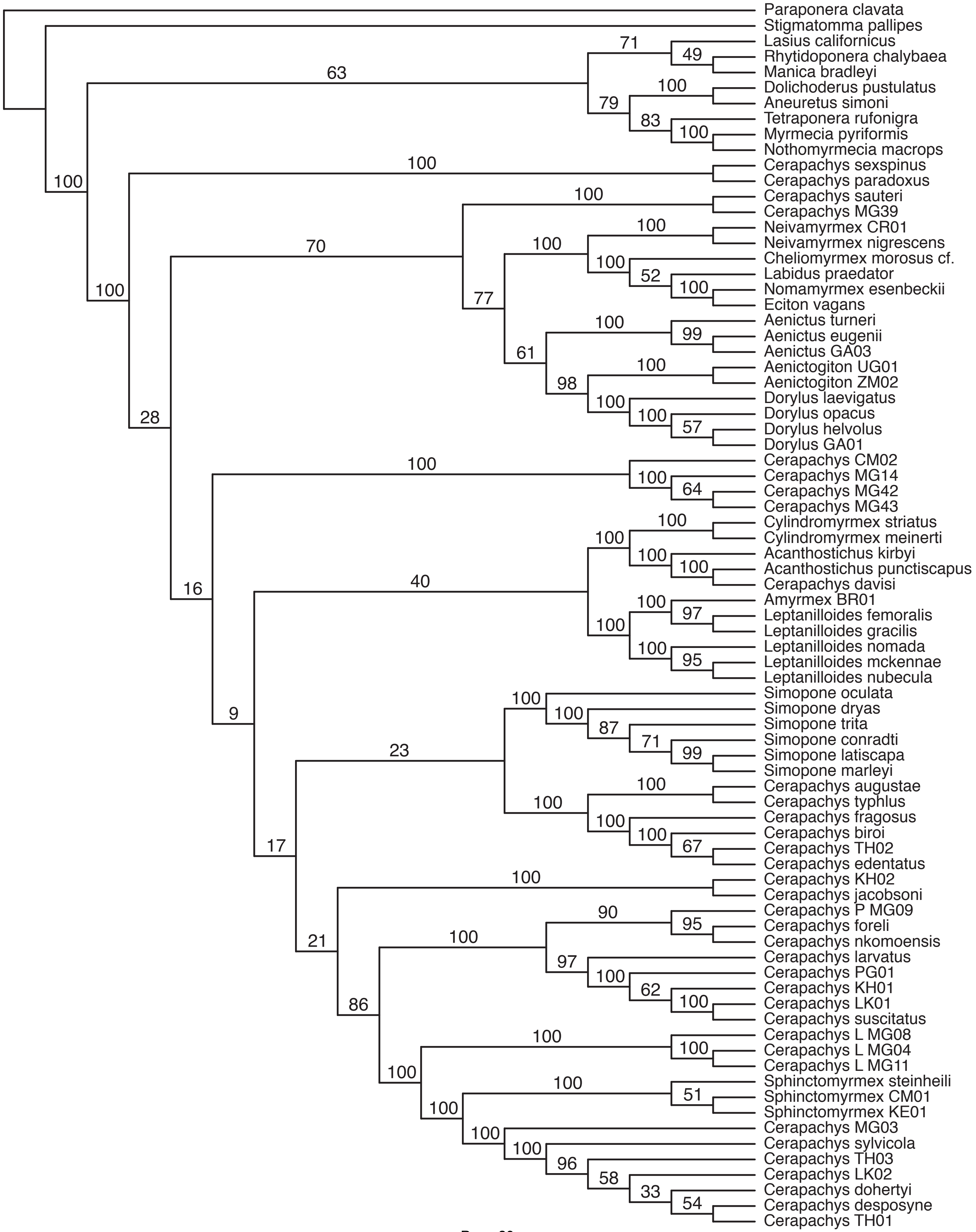

Dory73 ingroup only, pos3 coded ACTG, MB 50M, burnin=20M

Majority-rule consensus tree

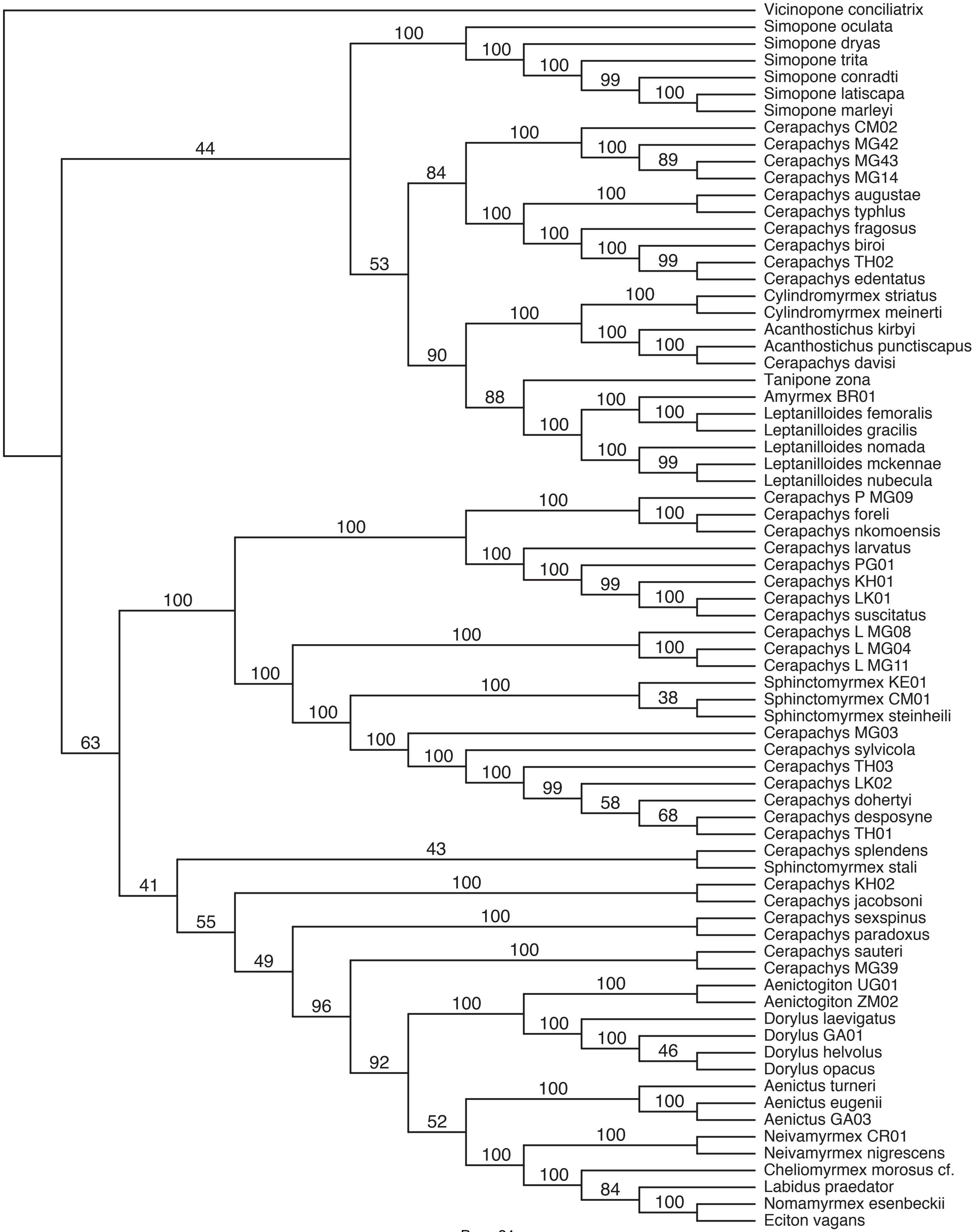

Dory73 ingroup only, all pos3s coded RY, MB 50M, burnin=20M, rooted like outgroup + ingroup analysis
Majority-rule consensus tree

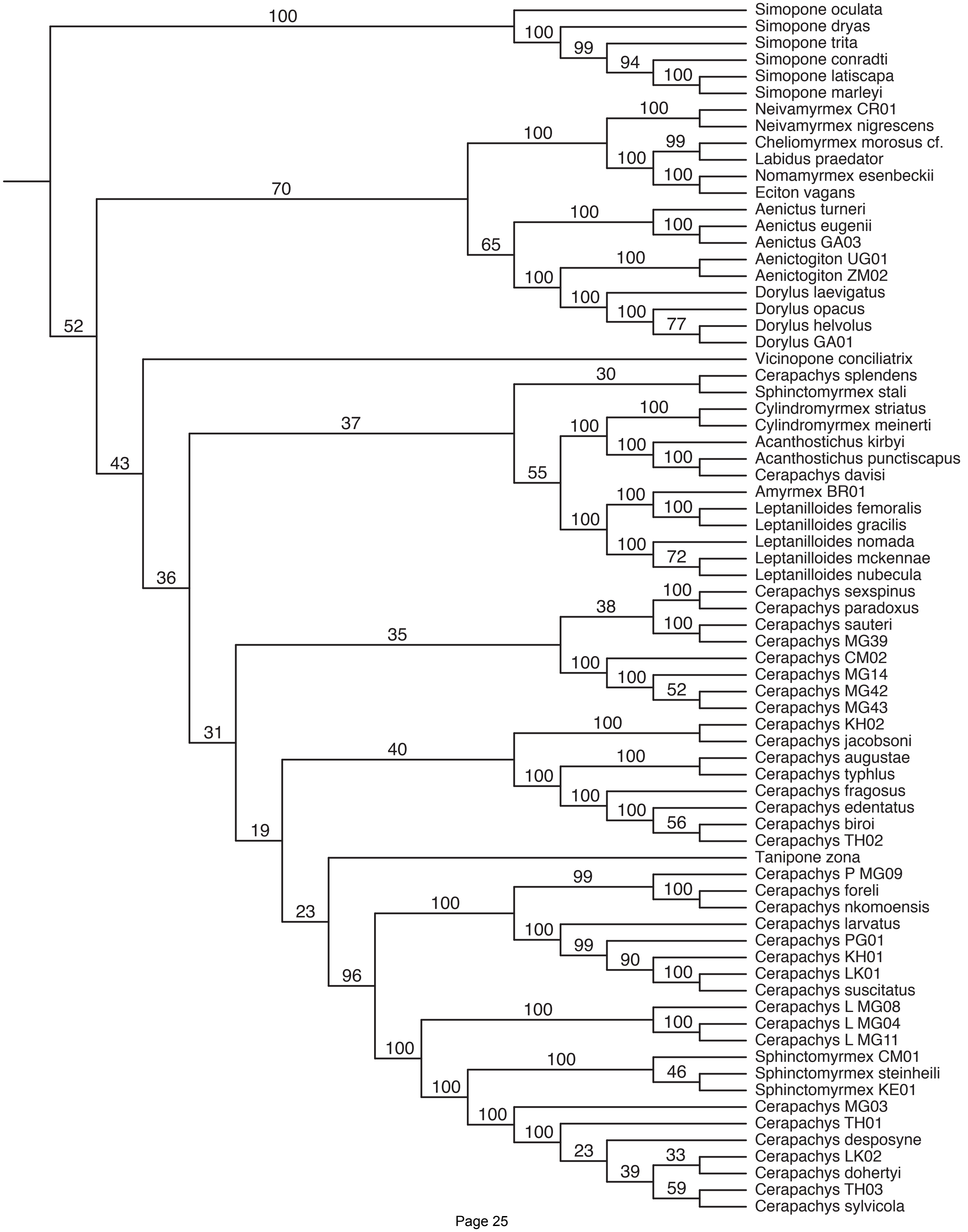

Dory69 ingroup only, all pos3s coded ACTG, MB 100M, burnin=80M, rooted like outgroup + ingroup analysis

Majority-rule consensus tree

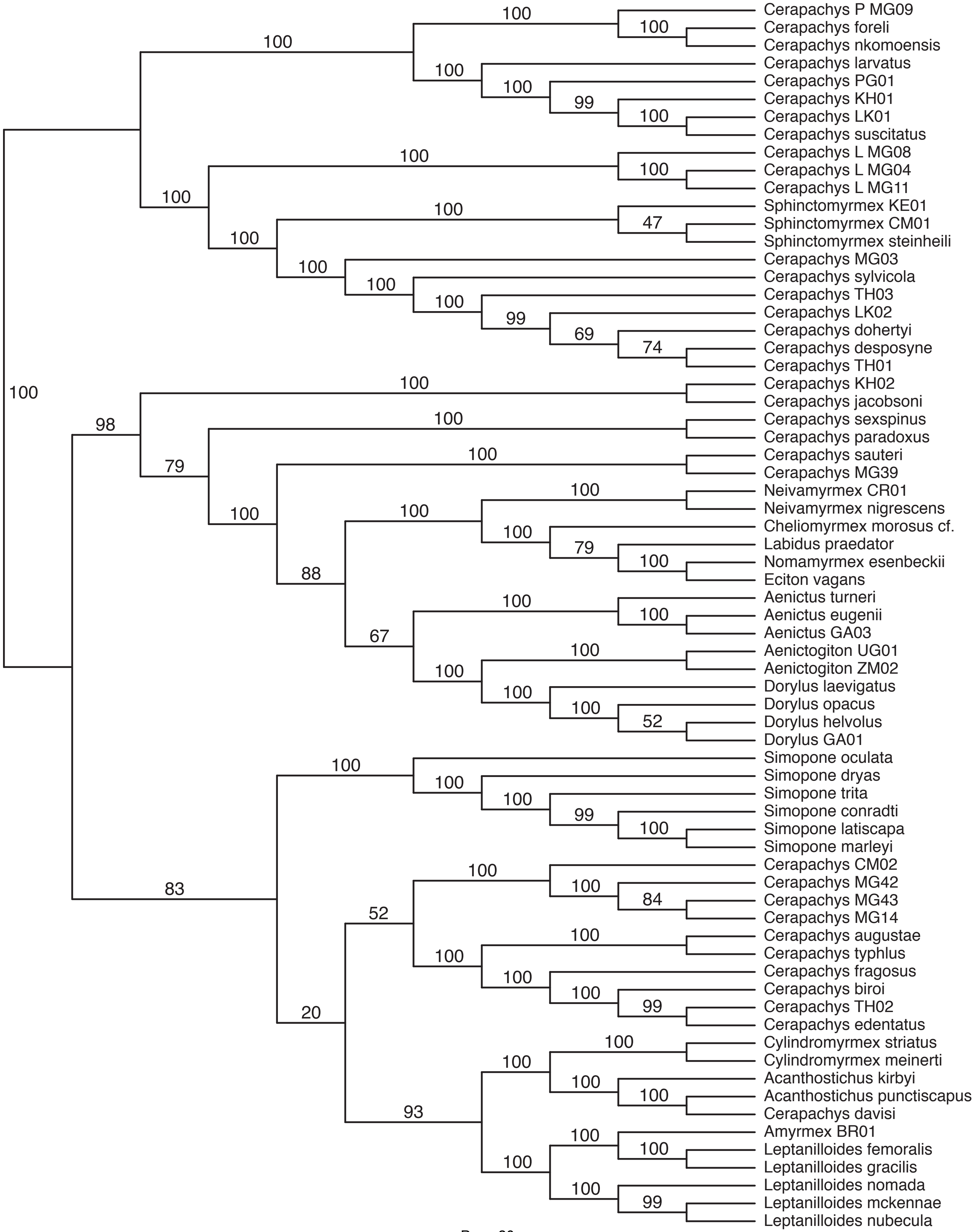

Dory69 ingroup only, all pos3s coded RY, MB 100M, burnin=25M, rooted like outgroup + ingroup analysis

Majority-rule consensus tree

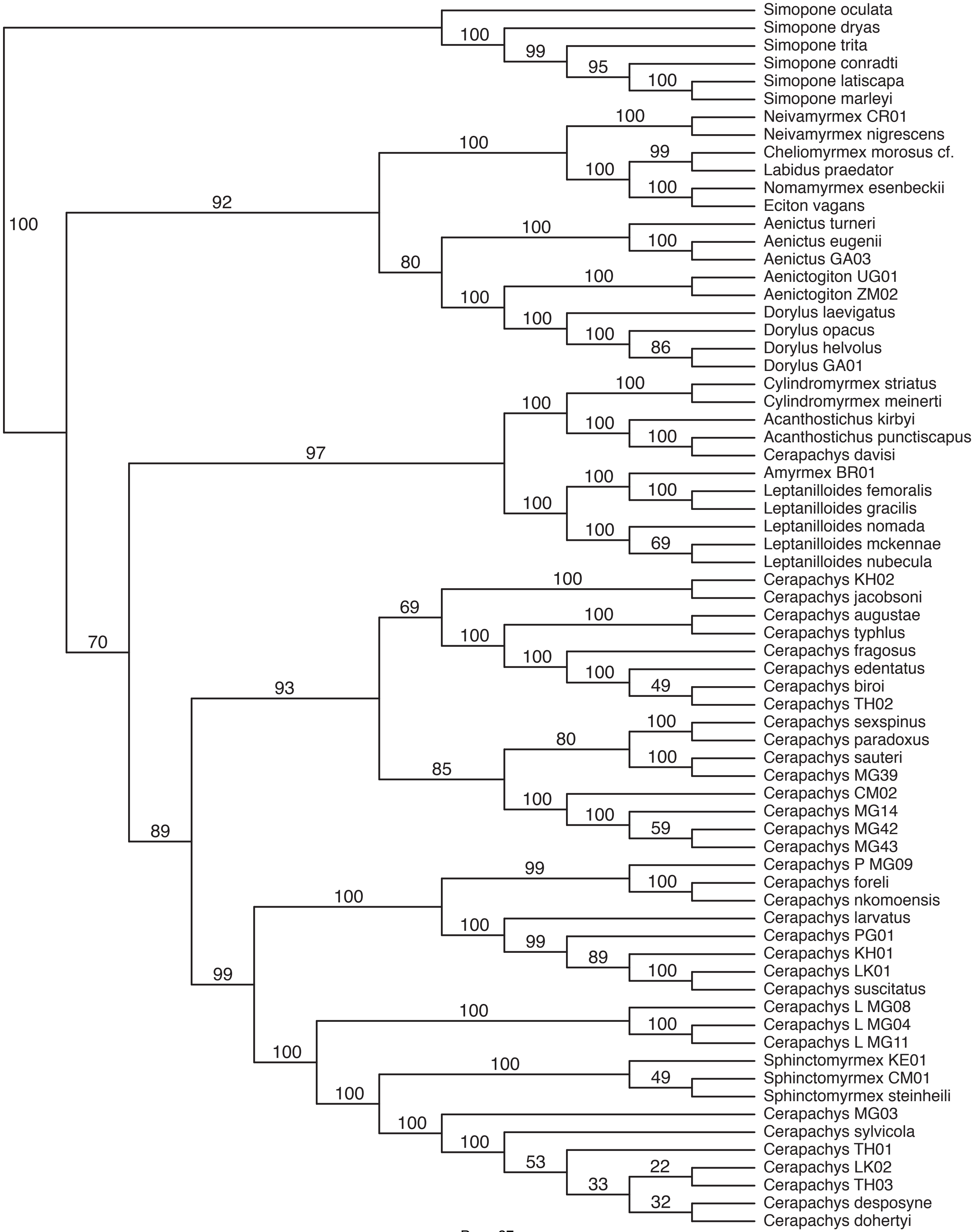

Dorylinae68, army ants excluded, Bayesian nuc models, 50M gens, burnin=30M

Majority-rule consensus tree

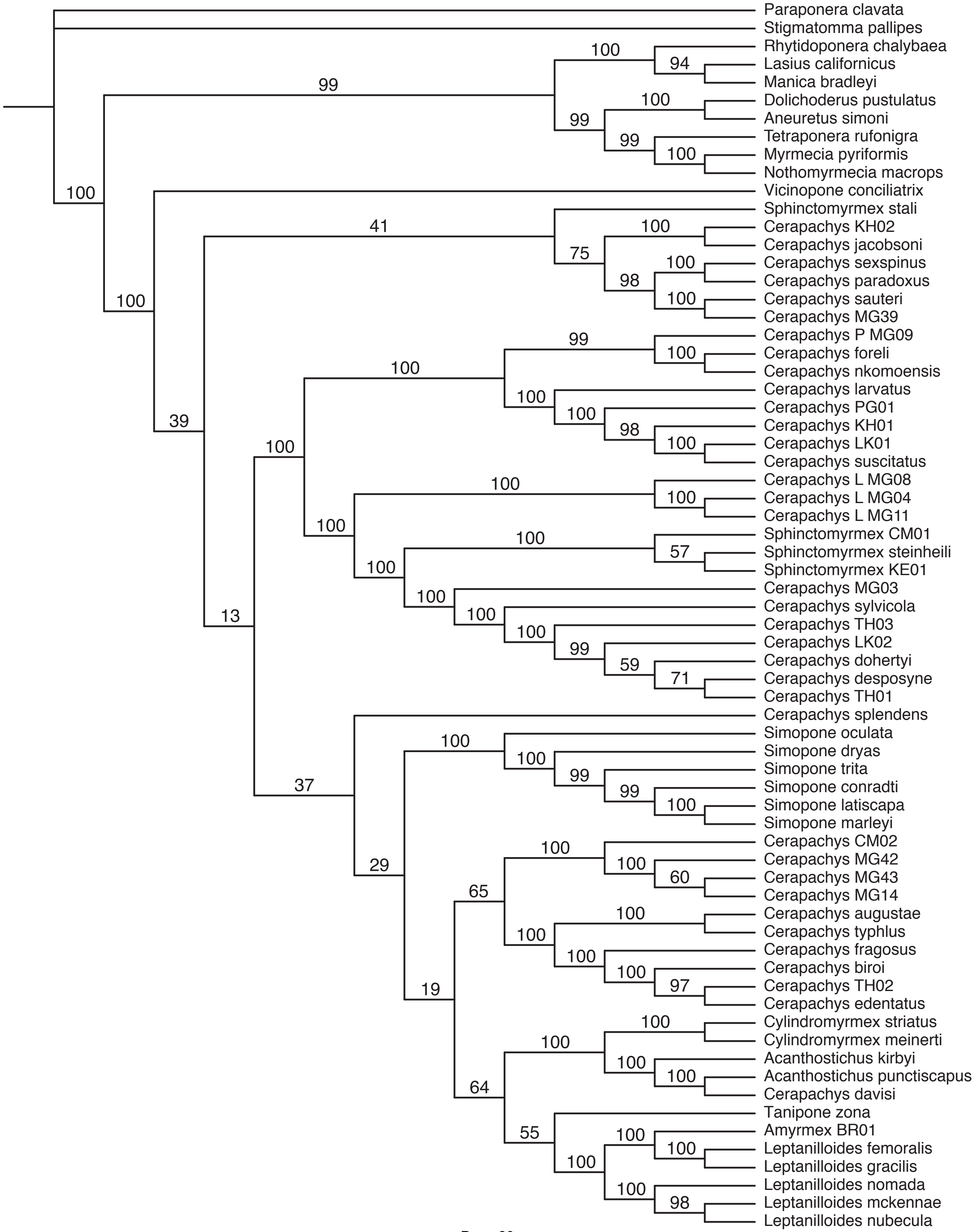

Dory83, 18S, Bayesian nuc model, 10M gens, burnin=1M

Majority-rule consensus tree

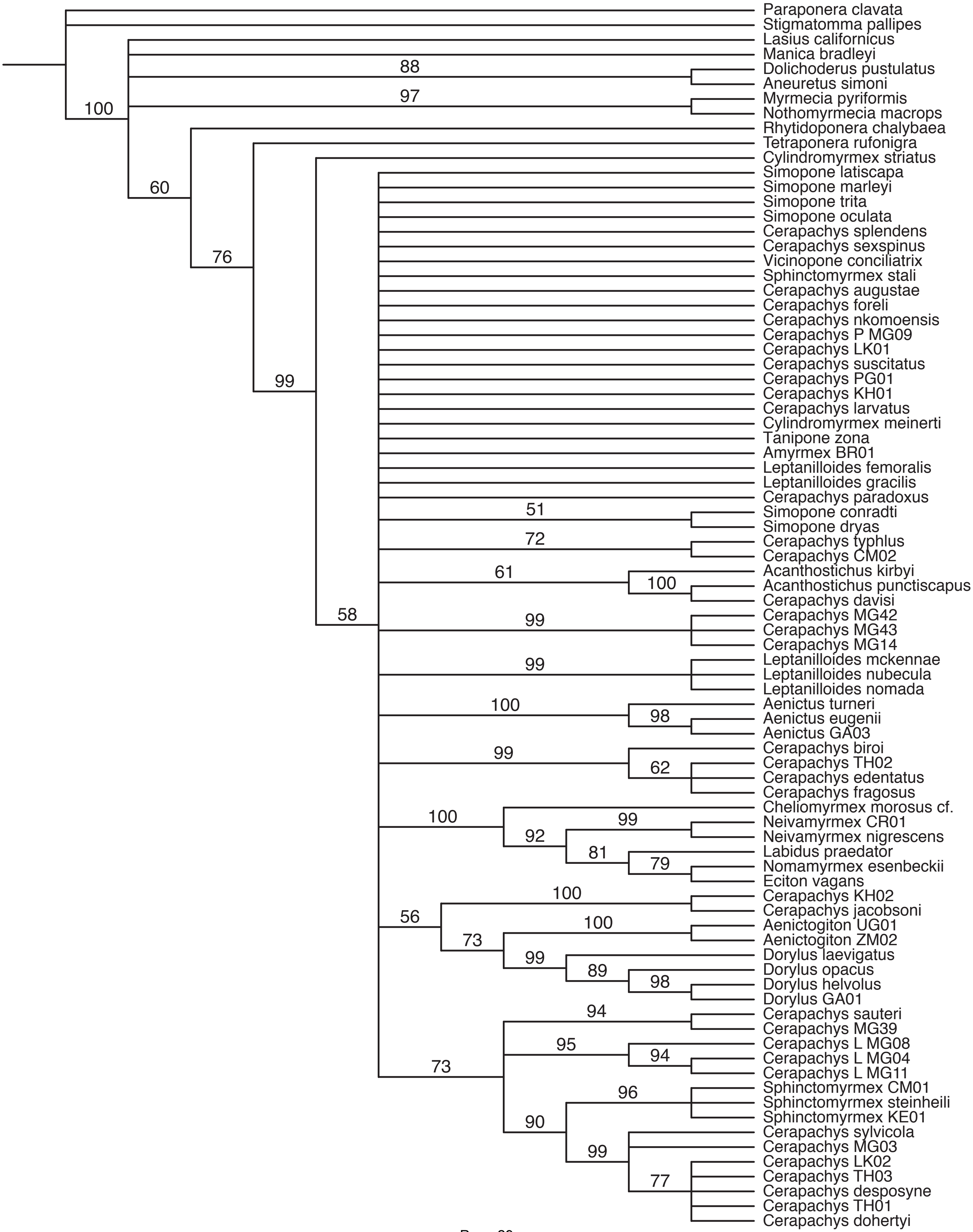

Dorylomorph 28S nuc model MB 10M gens burnin=1M

Majority-rule consensus tree

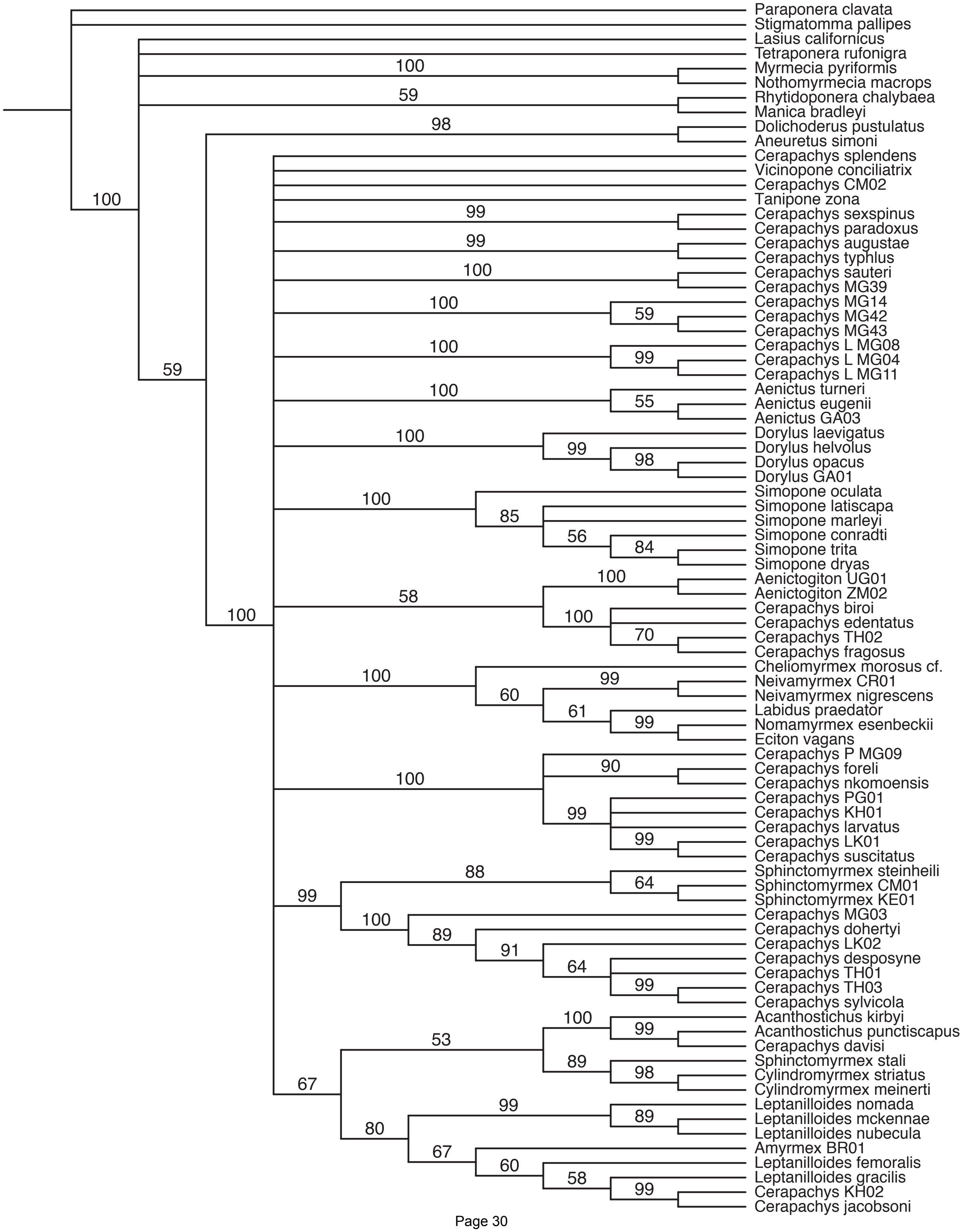

Dorylomorph abdA nuc models MB 10M gens burnin=1M

Majority-rule consensus tree

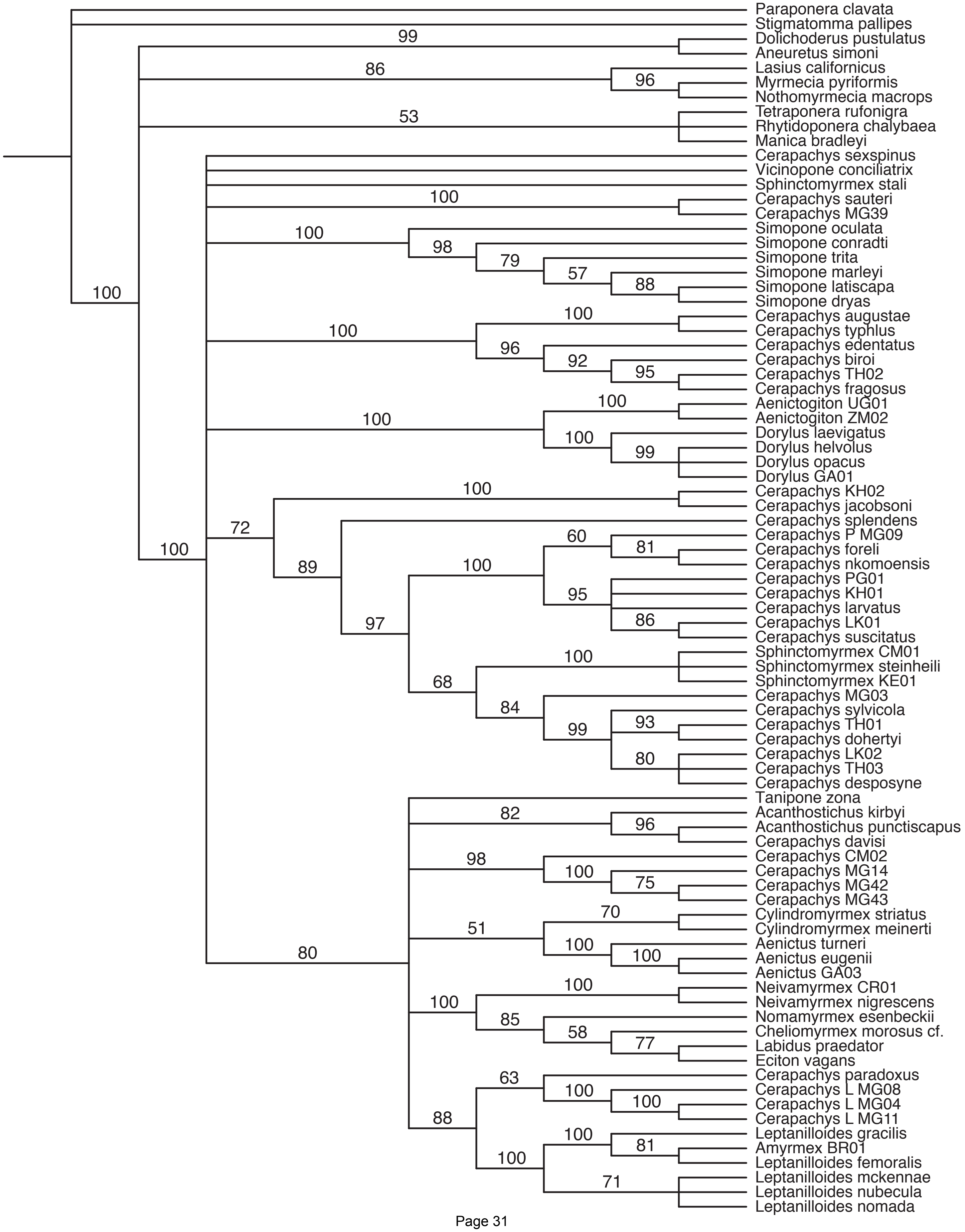

Dorylomorph 83 argK, Bayesian nuc model, 10M gens burnin=1M

Majority-rule consensus tree

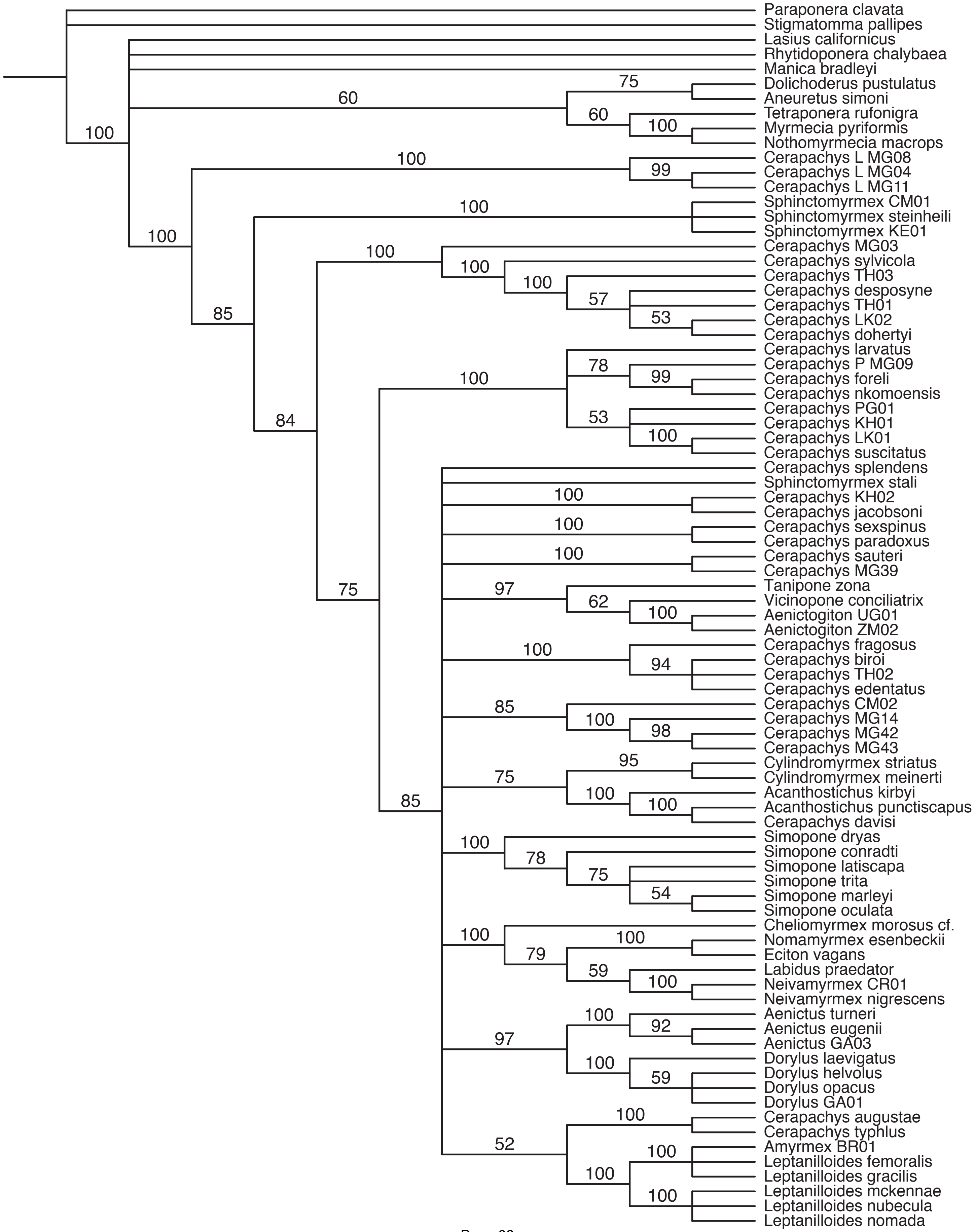

Dorylomorph 83 CAD, nuc model, mb 10M burnin=1M

Majority-rule consensus tree

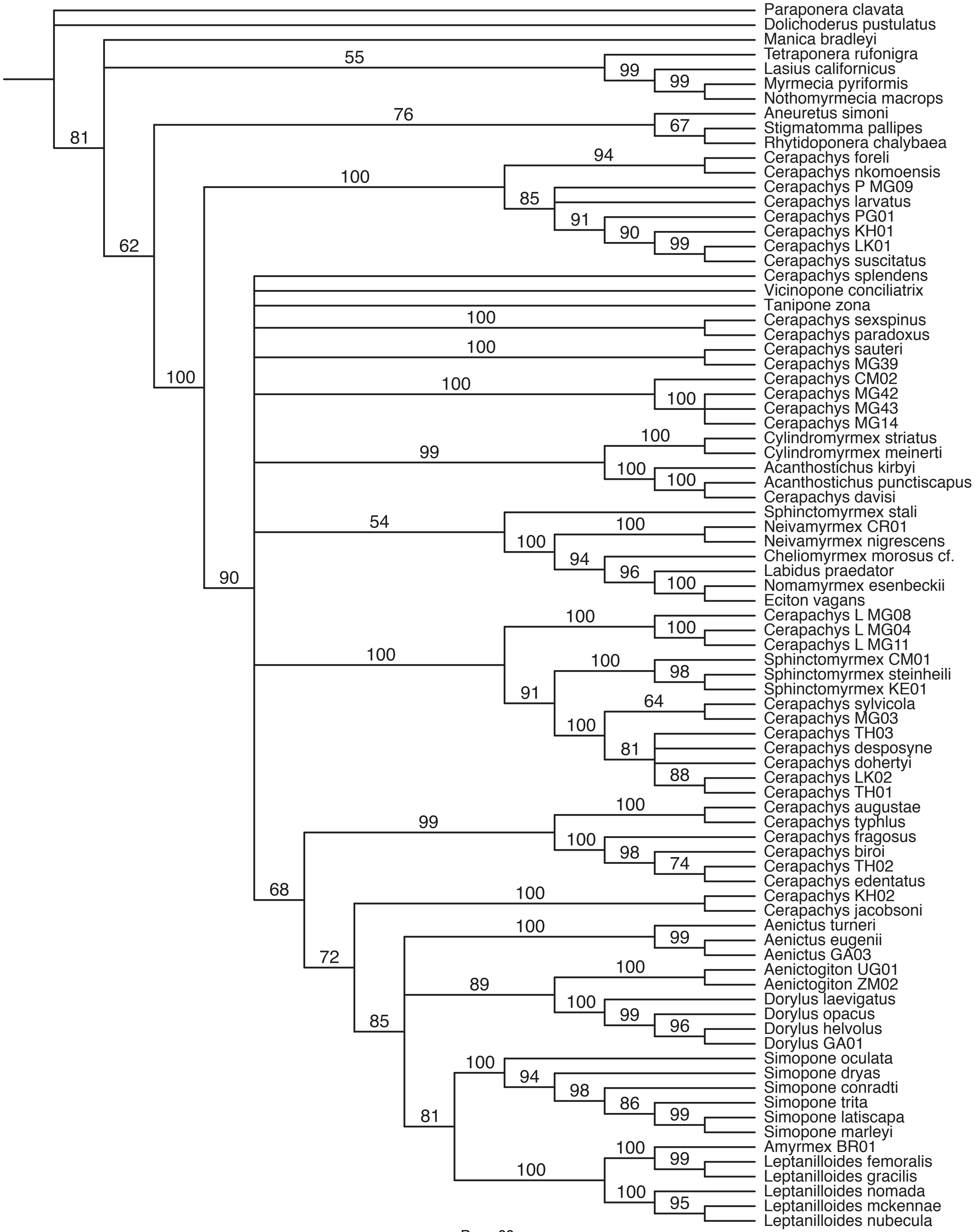

Dorylomorph 83 EF1aF1, nuc model, mb 10M burnin=1M

Majority-rule consensus tree

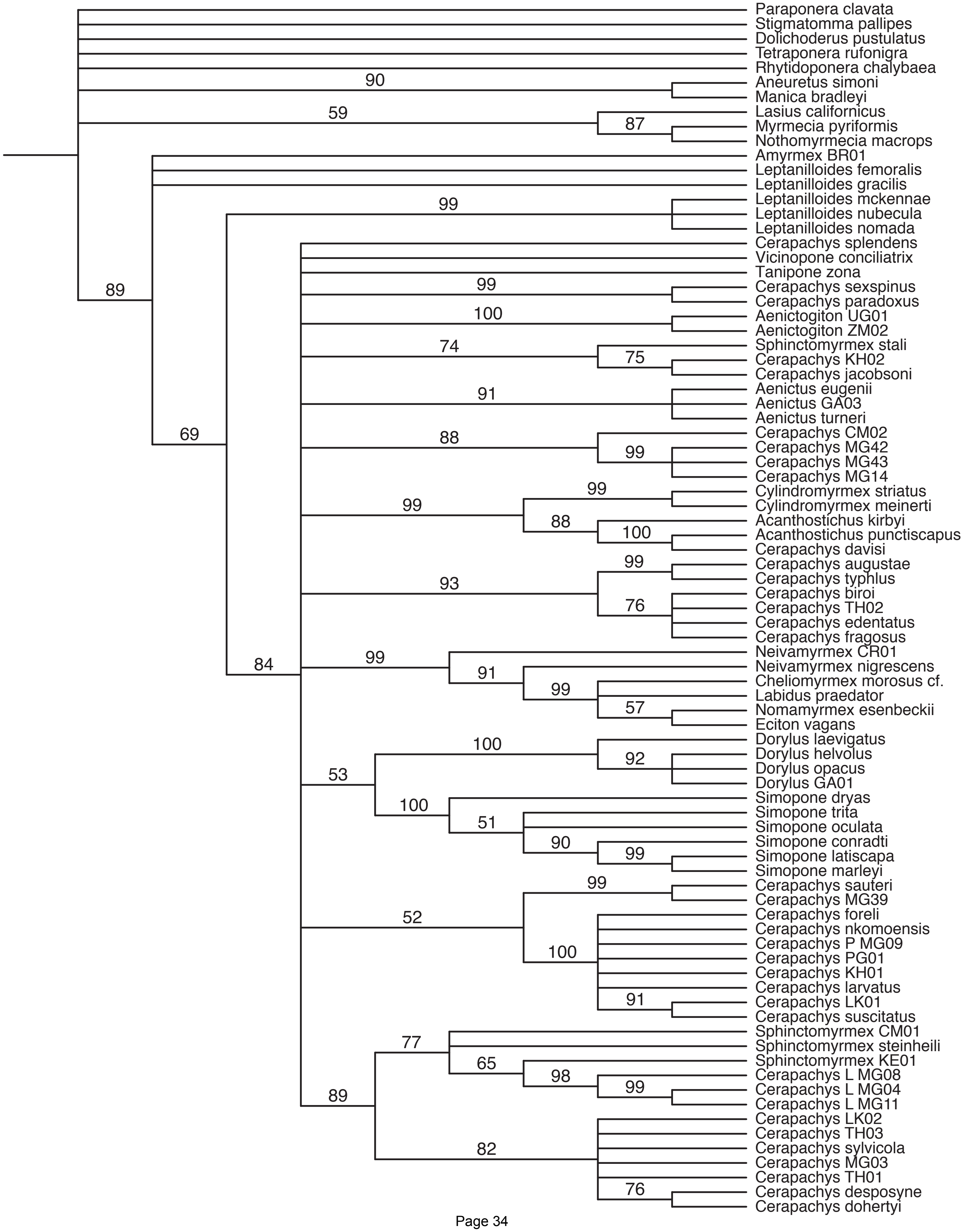

Dorylomorph 83 EF1aF2, nuc model, mb 10M burnin=1M

Majority-rule consensus tree

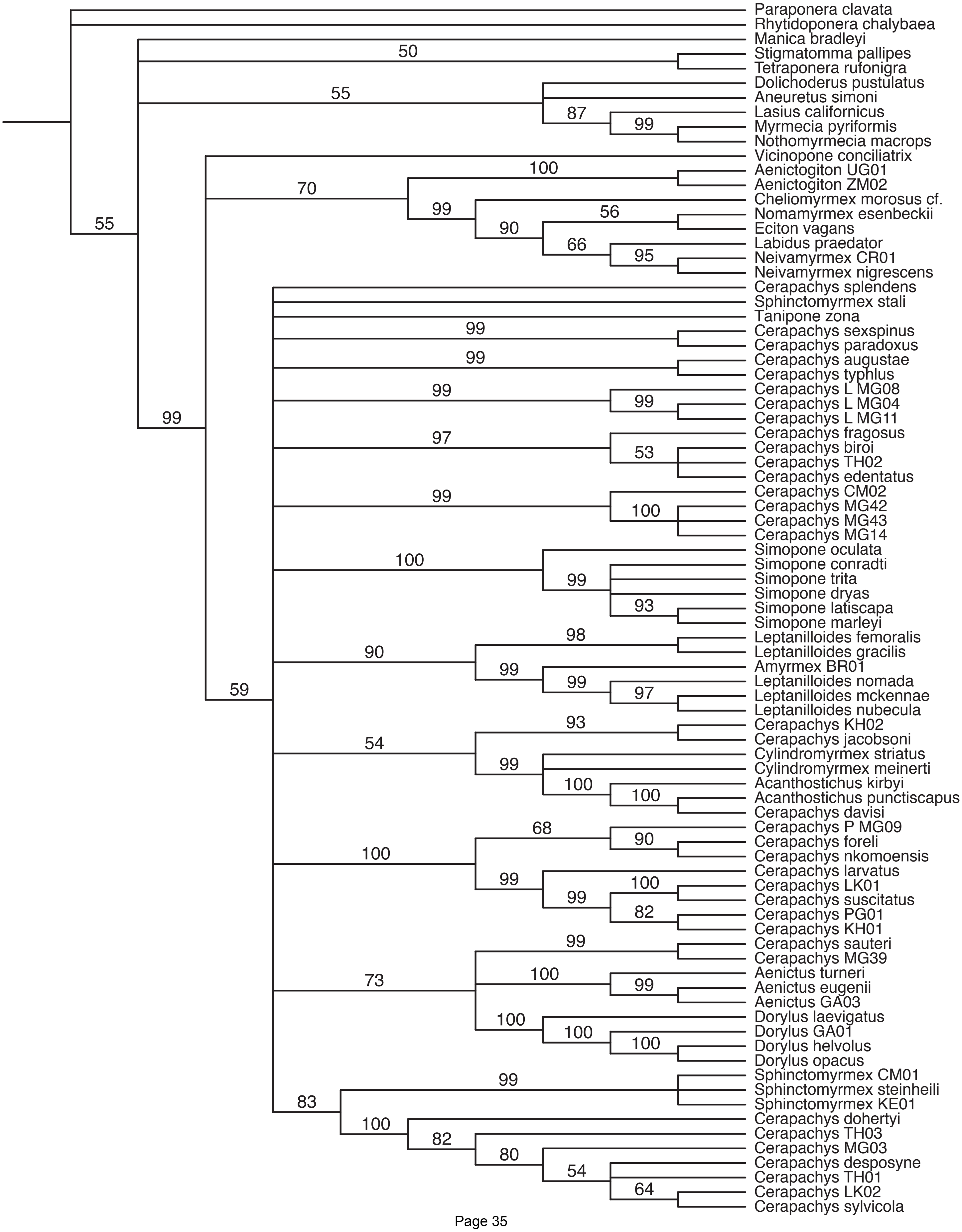

Dorylomorph 83 LWRh, nuc model, mb 10M burnin=1M

Majority-rule consensus tree

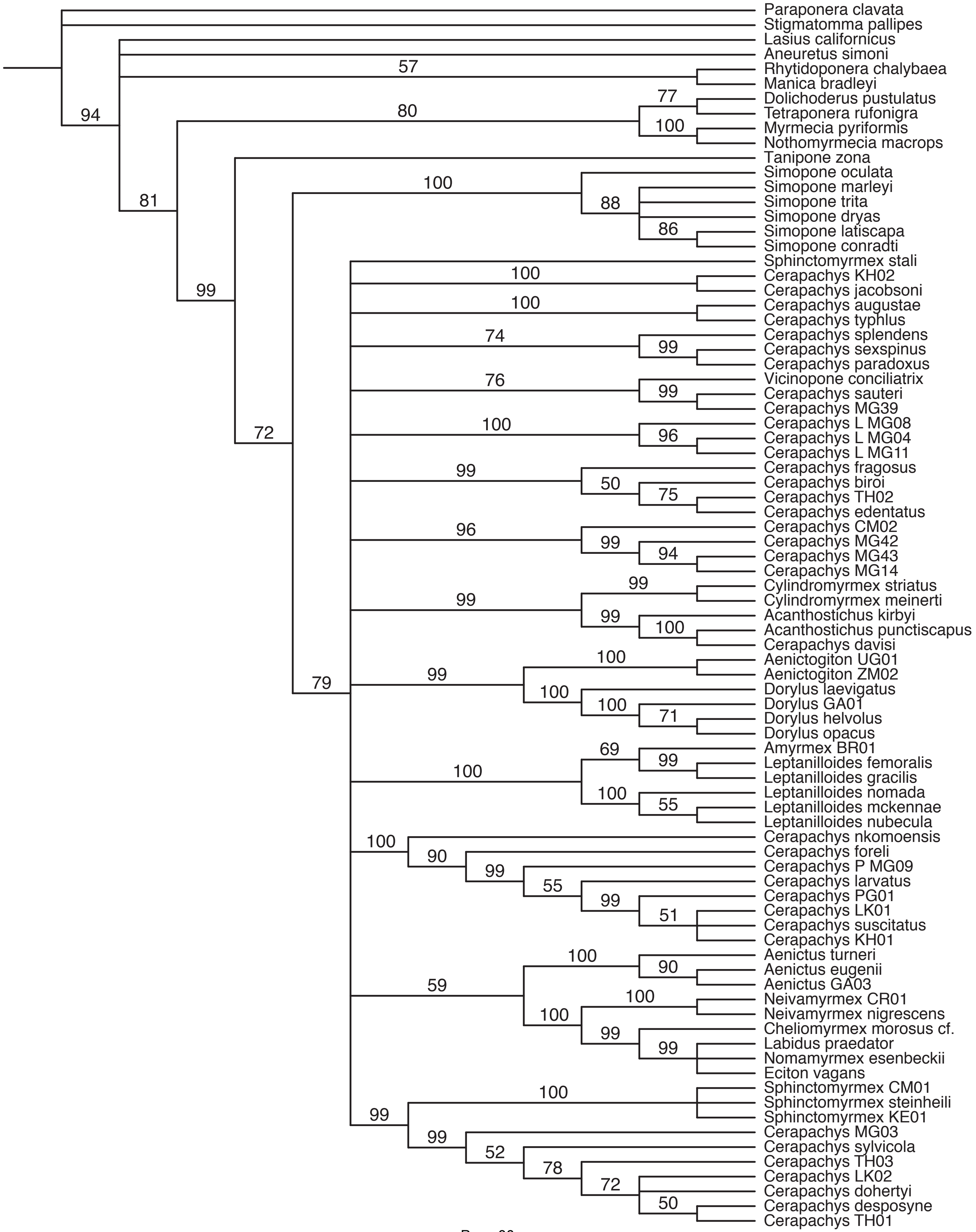

Dorylomorph 83, Top1 single gene, 20M gens, burnin=2M, 2 runs converged

Majority-rule consensus tree

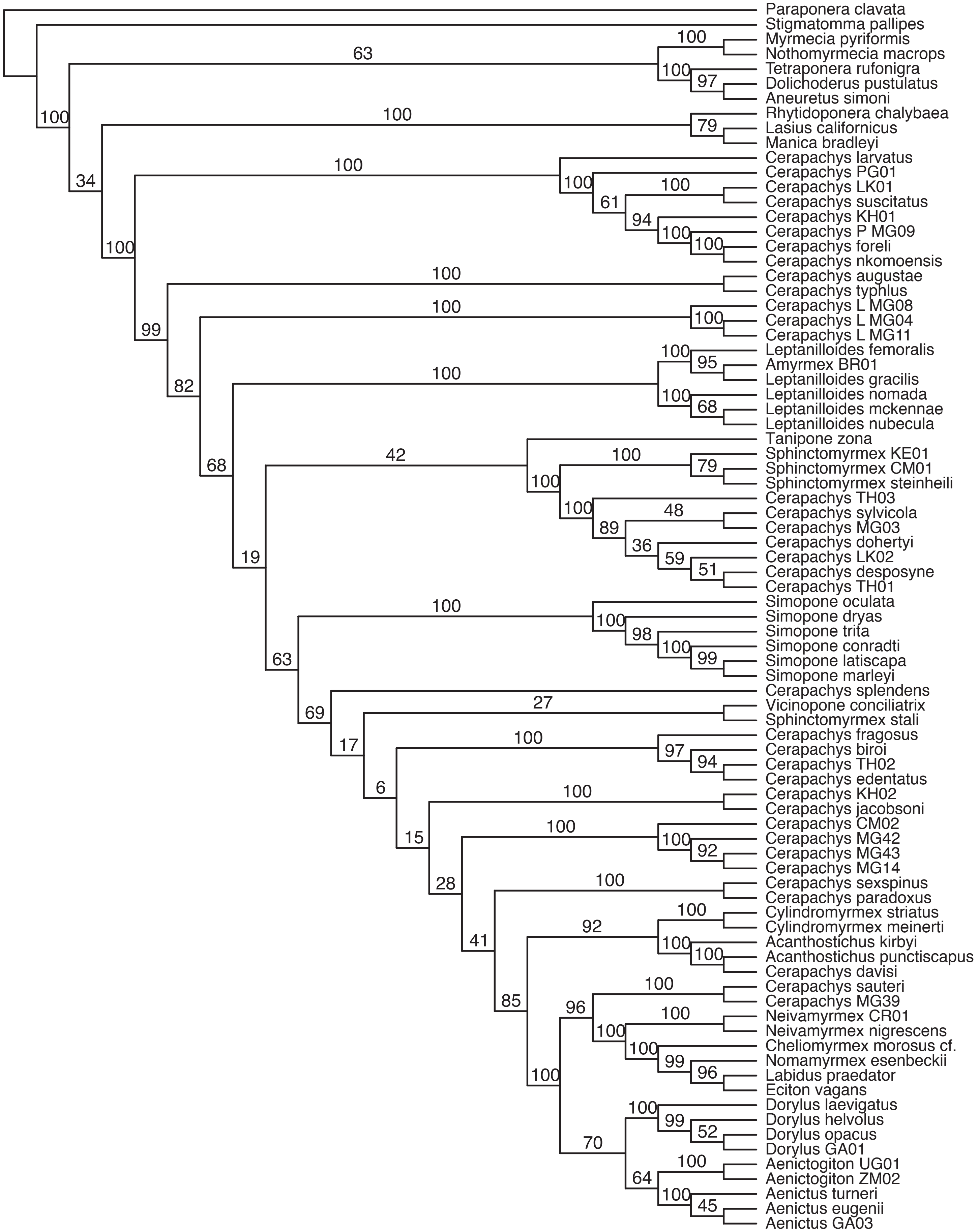

Dorylomorph 83 Ubx, nuc model, mb 10M burnin=1M

Majority-rule consensus tree

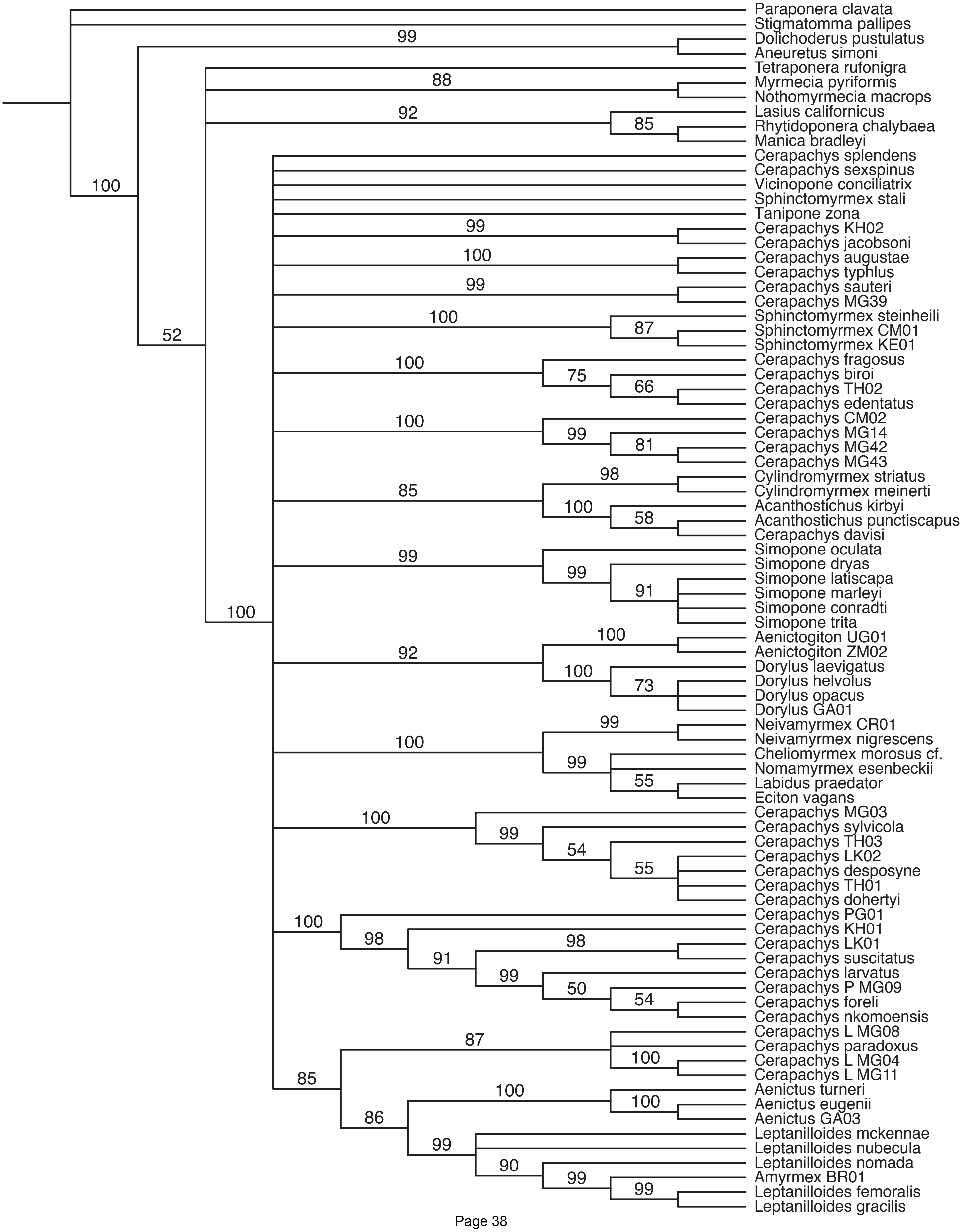

Dorylomorph 83 wg, nuc model, mb 10M burnin=1M

Majority-rule consensus tree

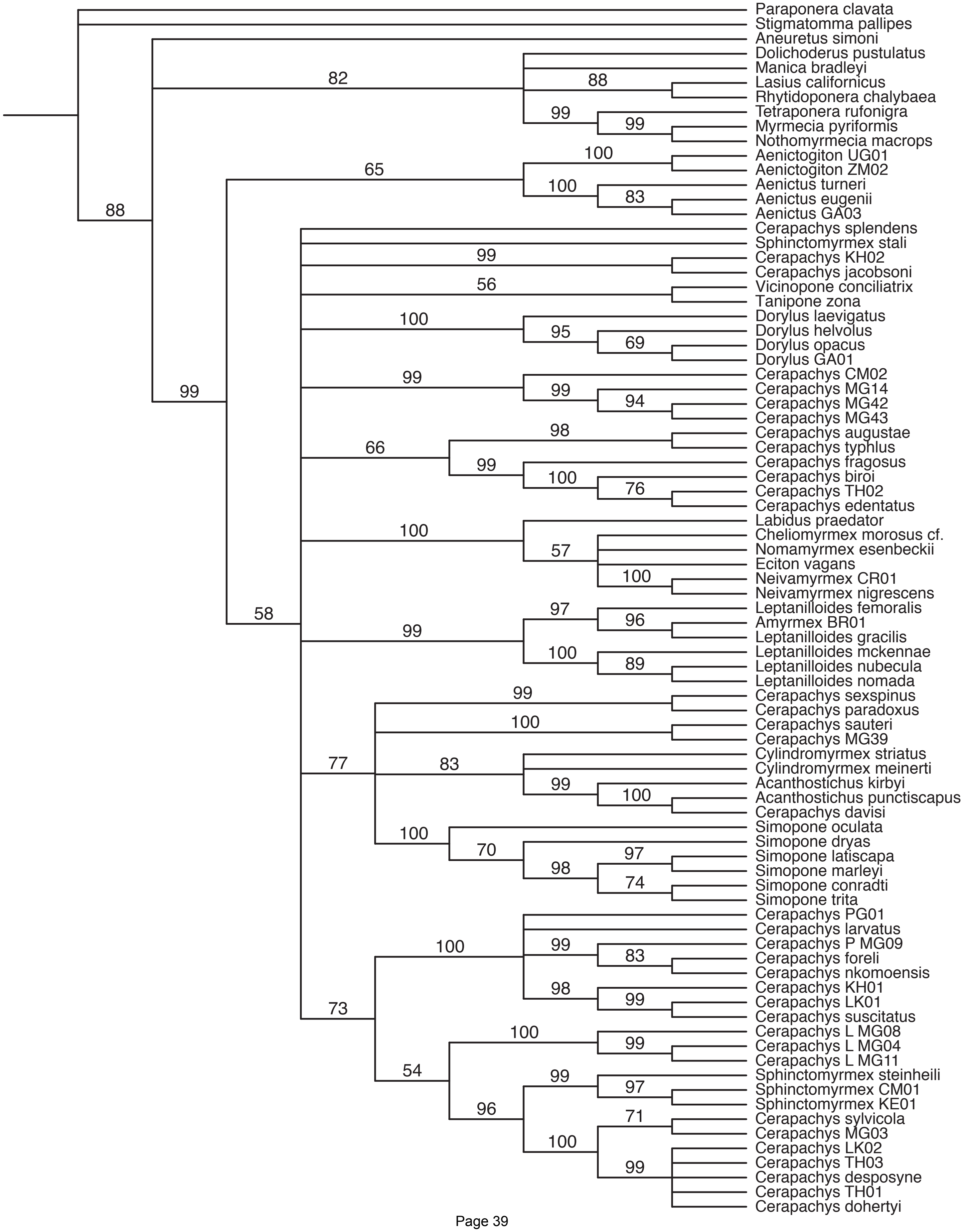

Supplement: Additional file 2 — Phylogenetic reconstructions from all data treatments. [file 1471-2148-14-93-S2.pdf]
